# Supplementary material for: Energy landscape remodeling mechanism of Hsp70-chaperone-accelerated protein folding
Source: Biophys J. 2021 Mar 19;120(10):1971–83. doi: 10.1016/j.bpj.2021.03.013 (PMC8204389; doi:10.1016/j.bpj.2021.03.013)
Supplement: Document S1. Figs. S1–S8 and Tables S1–S6 [file mmc1.pdf]

Biophysical Journal, Volume *120*

Supplemental information

Energy landscape remodeling mechanism of Hsp70-chaperone-accelerated protein folding

Jiajun Lu, Xiaoyi Zhang, Yichao Wu, Yuebiao Sheng, Wenfei Li, and Wei Wang

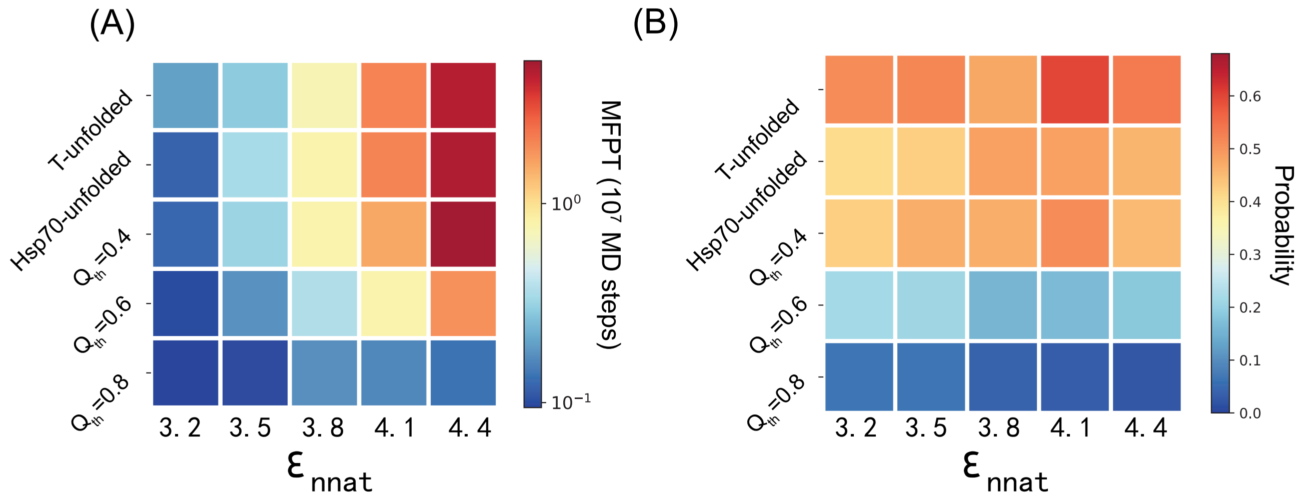

Figure S1. (MFPT of substrate hTRF1 folding (A) and populations of the frustrated folding pathway (B) calculated based on different simulation strategies and frustration extents for the hTRF1 with two pairs of non-native contacting interactions. T-unfolded: spontaneous folding starting from the initial structures prepared by thermal denaturation; Hsp70-unfolded: spontaneous folding starting from the initial structures prepared by unfolding action of the Hsp70; $Q_{\rm th}$ = 0.4, 0.6, and 0.8: Hsp70 mediated folding with the chaperone being removed when the Q score exceeds 0.4, 0.6, and 0.8, respectively.

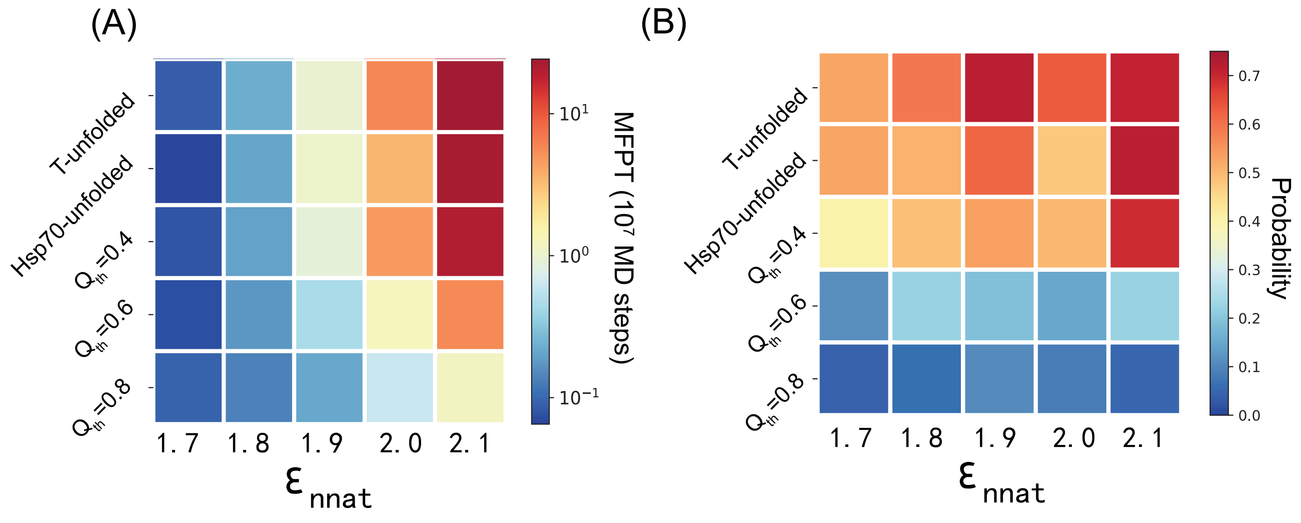

Figure S2. MFPT of substrate hTRF1 folding (A) and populations of the frustrated folding pathway (B) calculated based on different simulation strategies and frustration extents for the hTRF1 with 25 pairs of non-native contacting interactions.

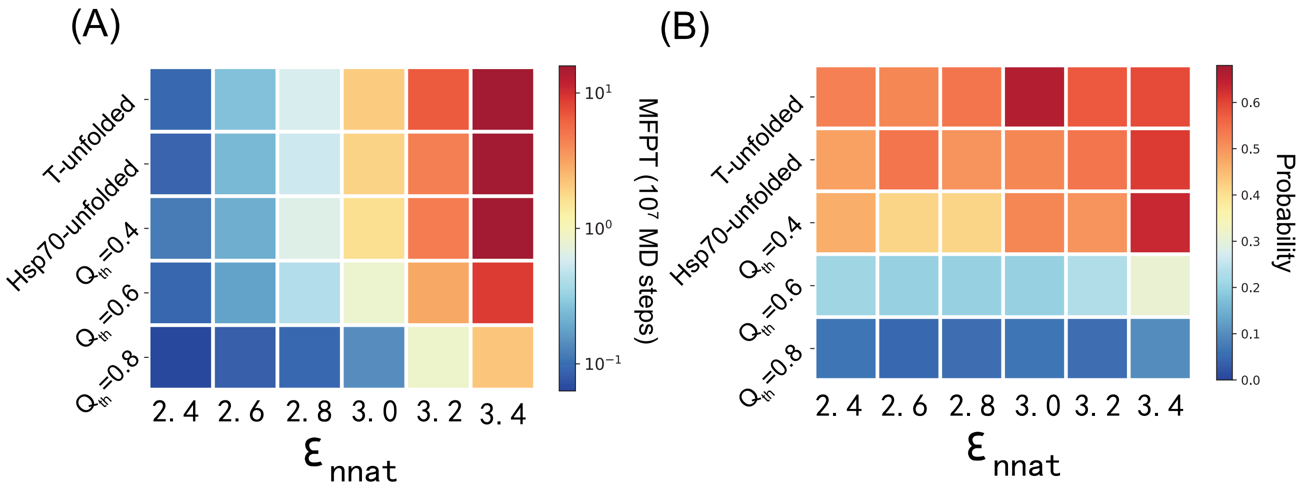

Figure S3. MFPT of substrate hTRF1 folding (A) and populations of the frustrated folding pathway (B) calculated based on different simulation strategies and frustration extents for the hTRF1 with the HP parameter C_{HP} set as 4.0.

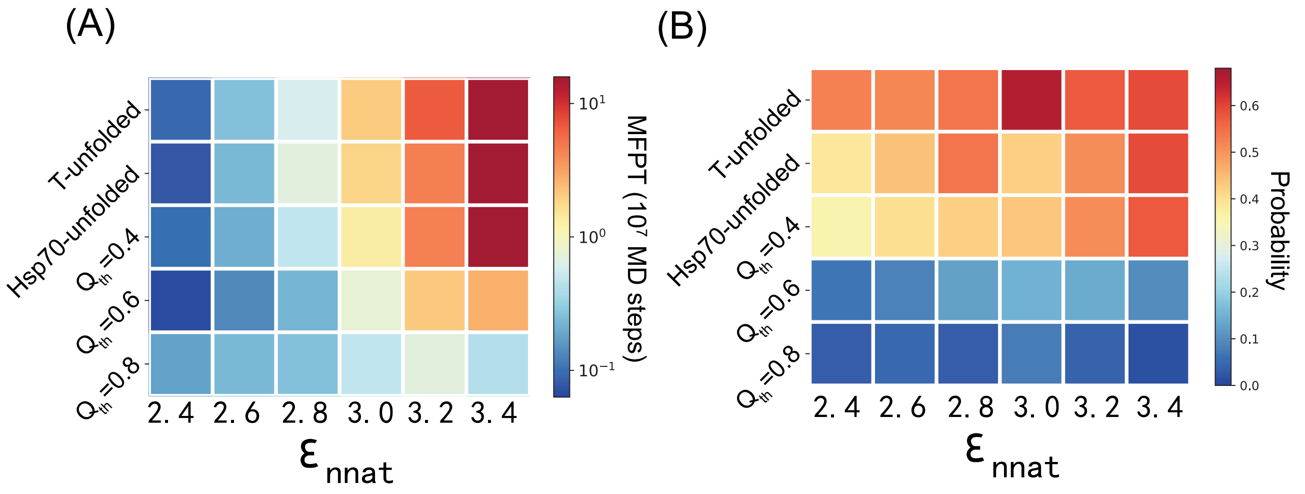

Figure S4. MFPT of substrate hTRF1 folding (A) and populations of the frustrated folding pathway (B) calculated based on different simulation strategies and frustration extents for the hTRF1 with the C_{HP} set as 6.0.

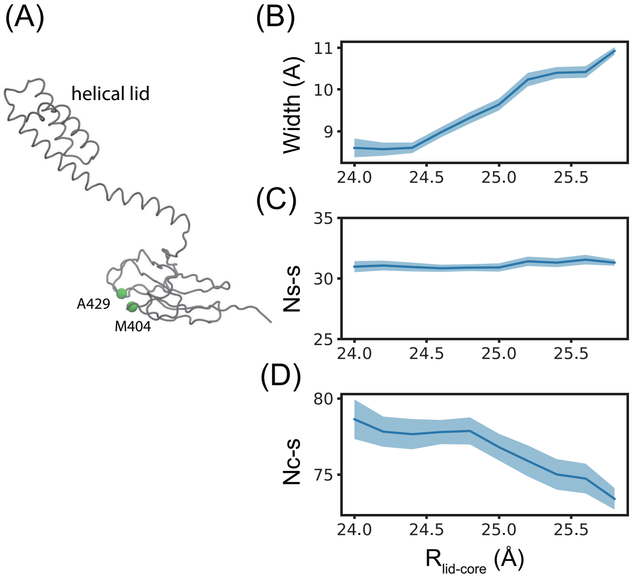

Figure S5. Effect of closed to open conformational change on the folding of the substrate hTRF1. (A) Illustrating structure of the substrate binding domain(SBD) of the Hsp70 with open conformation. (B) Average width of the substrate binding groove as a function of the reaction coordinate $R_{\mathrm{lid-core}}$. Here the reaction coordinate $R_{\mathrm{lid-core}}$ is defined by the distance between the centers of mass of the helical lid (S504 to A602) and the substrate binding core (V389 to A503) of the SBD, which was used to monitor the closed to open conformational change of the Hsp70. The width of the substrate binding groove was calculated based on the two labelled residues. (B) Average total number of the formed intra-molecule contacts for the substrate hTRF1. (C) The average number of the formed inter-molecule contacts between the substrate and the Hsp70.

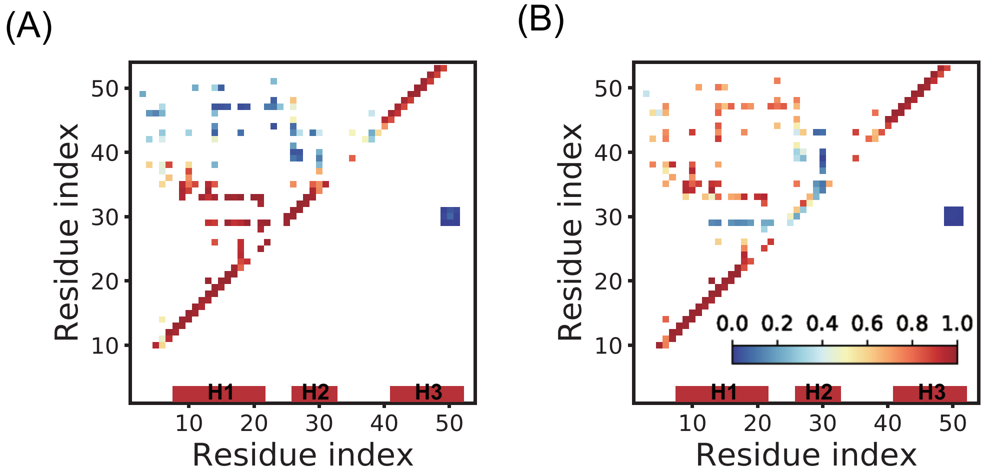

Figure S6. Structure features of the transition state ensembles of hTRF1 folding. (A,B) Probabilities of the contact formation at the transition state ensembles based on the simulations without (A) and with (B) the presence of the Hsp70 chaperone. The results for the native contacts are shown in the upper-left part, and the results for the nine residue pairs with non-native contacting interactions are shown in the lower-right part. One can see that the Hsp70 interactions tend to modulate the transition state structure of the substrate folding by favoring the formation of the native contacts between the H1 and H3.

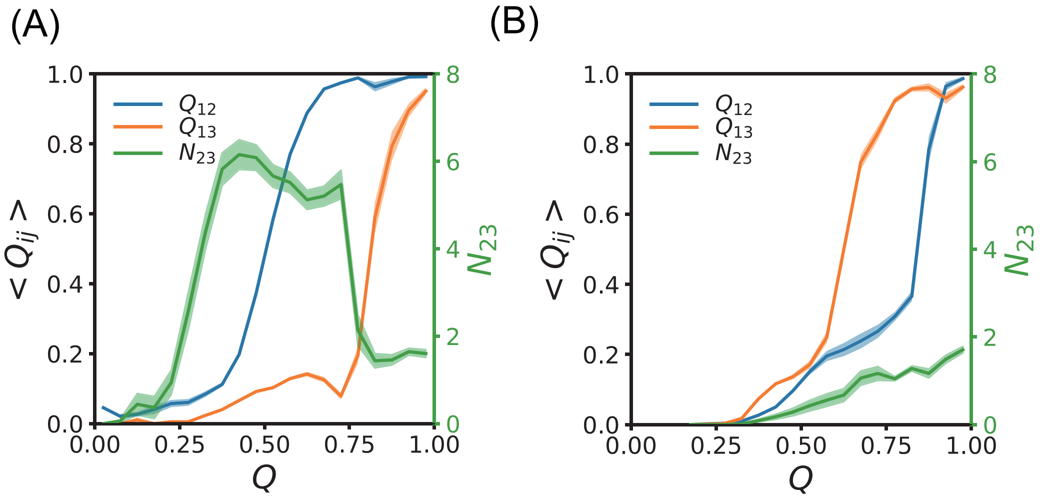

Figure S7. The average fraction of formed native contacts between the three helices (Q_{12}, Q_{13}) and the total number of the formed contacts between the H2 and H3 (N_{23}, right axis) as a function of the fraction of the formed total native contacts Q for the simulations of hTRF1 folding without (A) and with (B) the Hsp70 chaperon. Without Hsp70, the contacts between the H1 and H2 forms much earlier than those between the H1 and H3. Whereas with the presence of the Hsp70, the H1-H3 contacts (Q_{13}) can form at much earlier stage of the folding. Such results suggest that the Hsp70 tends to increase the population of the pathway via I_{13}. Consistent with the discussions in the main text, the formation of the non-native contacts between the H2 and H3 is much reduced with the presence of the Hsp70.

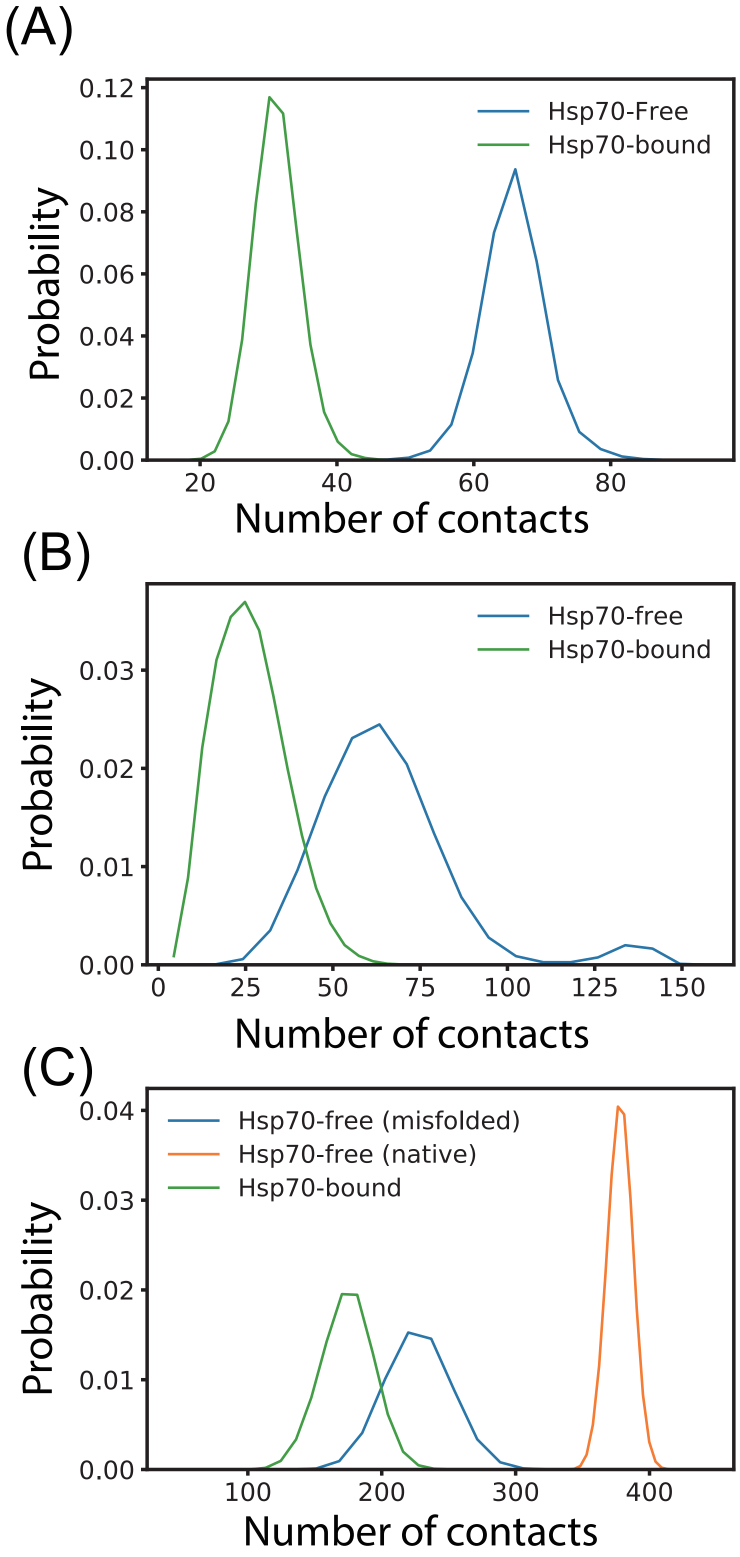

Figure S8. The distributions of the total intra-molecule contact numbers for substrate proteins hTRF1(A), SH3 (B), and RNase H (C) without Hsp70 binding (blue) and with the substrate bound to the closed form of the Hsp70 (green). The total contacts for the native state (blue) and misfolded state (orange) of the substrate RNase H were shown separately in (C).

Table S1. The scaling factor λ_{SB} controlling the interaction strengths of the structure-based interactions for the substrate proteins hTRF1, SH3, and RNase H. The ΔG_{NU} (or the population of unfolded state P_{U}) from simulations and experimental measurements (1–3).

Substrate	λ_{SB}	$P_{\mathrm{U}}^{\mathrm{simu.}}$	$P_{\mathrm{U}}^{\mathrm{exp.}}$
hTRF1	0.66	1%	1%
SH3	0.648	35%	38%
Substrate	λ_{SB}	$\Delta G_{UN}^{\mathrm{simu.}}$	$\Delta G_{\mathrm{UN}}^{\mathrm{exp.}}$
RNase H	0.60	10.2	9.7

Table S2. The residue pairs involved in the non-native contacting interactions for the three substrate proteins. The non-native contacting interactions were introduced for nine residue pairs.

hTRF1	I29-T49	I29-M50	I29-K51	I30-T49	I30-M50	I30-K51	L31-T49	L31-M50	L31-K51
SH3	A11-Q23	A11-I24	A11-L25	T12-Q23	T12-I24	T12-L25	A13-Q23	A13-I24	A13-L25
RNase H	I25-A109	I25-A110	I25-L111	L26-A109	L26-A110	L26-L111	R27-A109	R27-A110	R27-L111

Table S3. The residue pairs involved in the non-native contacting interactions for the hTRF1. The non-native contacting interactions were introduced for 2 or 25 residue pairs.

2 pairs	I30-M50	L31-M50							
25 pairs	K28-R48	K28-T49	K28-M50	K28-K51	K28-K52	I29-R48	I29-T49	I29-M50	I29-K51
	I29-K52	L30-R48	L30-T49	L30-M50	L30-K51	L30-K52	L31-R48	L31-T49	L31-M50
	L31-K51	L31-K52	H32-R48	H32-T49	H32-M50	H32-K51	H32-K52		

Table S4. The parameters set ΔV_s and Δ_s corresponding to the ADP state (closed conformation is much more stable than the open conformation) and ATP state (open conformation is much more stable than the closed conformation).

State	ΔV_s	Δ_s
ADP	-60	125
ATP	35	125

Table S5. The parameters used in the hydrophobic interactions for the chaperone-substrate interactions(4)

Amino acid	Ala	Arg	Asn	Asp	Cys	Gln	Glu	Gly	His	Ile
$\epsilon_{\mathrm{HP}}^{A}$	0.42	-1.37	-0.82	-1.05	2.09	-0.30	-0.87	0.00	0.18	2.45
Amino acid	Leu	Lys	Met	Phe	Pro	Ser	Thr	Trp	Tyr	Val
$\epsilon_{\mathrm{HP}}^{A}$	2.31	-1.35	1.67	2.43	0.98	-0.05	0.35	3.06	1.31	1.66

Table S6. The parameters used in the excluded volume interactions for the chaperone-substrate interactions taken from Ref. (5). The original values were scaled by 0.9.

Amino acid	Ala	Arg	Asn	Asp	Cys	Gln	Glu	Gly	His	Ile
σ(Å)	4.50	5.94	5.13	5.04	4.95	5.40	5.31	4.05	5.49	5.58
Amino acid	Leu	Lys	Met	Phe	Pro	Ser	Thr	Trp	Tyr	Val
σ(Å)	5.58	5.76	5.58	5.76	5.04	4.68	5.04	6.12	5.85	5.31

Supporting References

1. A Sekhar, R Rosenzweig, G Bouvignies, LE Kay, Mapping the conformation of a client protein through the hsp70 functional cycle. *Proc. Natl. Acad. Sci. U.S.A.* **112**, 10395–10400 (2015).
2. JH Lee, et al., Heterogeneous binding of the sh3 client protein to the dnak molecular chaperone. *Proc. Natl. Acad. Sci. U.S.A.* **112**, E4206–E4215 (2015).
3. GM Spudich, EJ Miller, S Marqusee, Destabilization of the escherichia coli rnase h kinetic intermediate: switching between a two-state and three-state folding mechanism. *J. Mol. Biol.* **335**, 609–618 (2004).
4. T Tanaka, N Hori, S Takada, How co-translational folding of multi-domain protein is affected by elongation schedule: Molecular simulations. *PLOS Comput. Biol.* **11** (2015).
5. YC Kim, G Hummer, Coarse-grained models for simulations of multiprotein complexes: application to ubiquitin binding. *J. Mol. Biol.* **375**, 1416–1433 (2008).
