# Supplementary material for: Energy landscape remodeling mechanism of Hsp70-chaperone-accelerated protein folding
Source: Biophys J. 2021 Mar 19;120(10):1971–83. doi: 10.1016/j.bpj.2021.03.013 (PMC8204389; doi:10.1016/j.bpj.2021.03.013)
Supplement: Document S2. Article plus supporting material [file mmc2.pdf]

Biophysical Journal
Article

Biophysical Society

Energy landscape remodeling mechanism of Hsp70-chaperone-accelerated protein folding

Jiajun Lu,[1] Xiaoyi Zhang,[1] Yichao Wu,[1] Yuebiao Sheng,[1] Wenfei Li,[1,*] and Wei Wang[1,*]
[1]Department of Physics, National Laboratory of Solid State Microstructure, Collaborative Innovation Center of Advanced Microstructures, Nanjing University, Nanjing, China

ABSTRACT Hsp70 chaperone is one of the key protein machines responsible for the quality control of protein production in cells. Facilitating in vivo protein folding by counteracting misfolding and aggregation is the essence of its biological function. Although the allosteric cycle during its functional actions has been well characterized both experimentally and computationally, the mechanism by which Hsp70 assists protein folding is still not fully understood. In this work, we studied the Hsp70-mediated folding of model proteins with rugged energy landscape by using molecular simulations. Different from the canonical scenario of Hsp70 functioning, which assumes that folding of substrate proteins occurs spontaneously after releasing from chaperones, our results showed that the substrate protein remains in contacts with the chaperone during its folding process. The direct chaperone-substrate interactions in the open conformation of Hsp70 tend to shield the substrate sites prone to form non-native contacts, which therefore avoids the frustrated folding pathway, leading to a higher folding rate and less probability of misfolding. Our results suggest that in addition to the unfoldase and holdase functions widely addressed in previous studies, Hsp70 can facilitate the folding of its substrate proteins by remodeling the folding energy landscape and directing the folding processes, demonstrating the foldase scenario. These findings add new, to our knowledge, insights into the general molecular mechanisms of chaperone-mediated protein folding.

SIGNIFICANCE Protein folding is one of the key molecular events in cells. Failure to fold properly may lead to nonproductive aggregation and various of neurodegenerative diseases. Hsp70 chaperone plays crucial roles in the protein folding under complex cellular environment. Although it is clear that the Hsp70 chaperones can prevent protein aggregation and increase folding efficiency, whether the chaperones can be directly involved in the folding process is still unclear. Here, by performing molecular dynamics simulations, we showed that the Hsp70 chaperone can direct the folding procedure of the substrate protein by remodeling the folding energy landscape and avoiding the frustrated folding pathway. Such an effect relies on the direct interactions between the substrate and chaperone during folding, suggesting a foldase mechanism.

INTRODUCTION

Although most of our current knowledge on protein folding has been accumulated based on in vitro biochemical studies, protein folding in cells often involves a more complicated microenvironment, such as molecular crowding and oxidative stress, which tends to increase the risk of misfolding and aggregation (1). Accordingly, a large fraction of proteins cannot fold spontaneously without assistance from folding machines. Cells have evolved various kinds of chaperones to aid in robust folding in such complex environments and prevent nonproductive misfolding and aggregation, which is essential for the integrity of molecular events in the life cycle of cells (2–6). Revealing the underlying molecular mechanism of chaperone-mediated protein folding is one of the central focuses in protein folding studies.

Chaperone-mediated protein folding typically involves the steps of substrate exchange, co-chaperone binding and unbinding, ATP binding and hydrolysis, and allosteric motions of the chaperone molecules (7–9), which makes in vivo studies of protein folding much more difficult than that of spontaneous folding under dilute conditions. As a consequence, accurate molecular mechanisms of how the molecular chaperones assist in vivo protein folding remain

Submitted October 5, 2020, and accepted for publication March 12, 2021.
*Correspondence: wfli@nju.edu.cn or wangwei@nju.edu.cn
Jiajun Lu and Xiaoyi Zhang contributed equally to this work.
Editor: Yuji Sugita.
https://doi.org/10.1016/j.bpj.2021.03.013
© 2021 Biophysical Society.
This is an open access article under the CC BY license (http://creativecommons.org/licenses/by/4.0/).

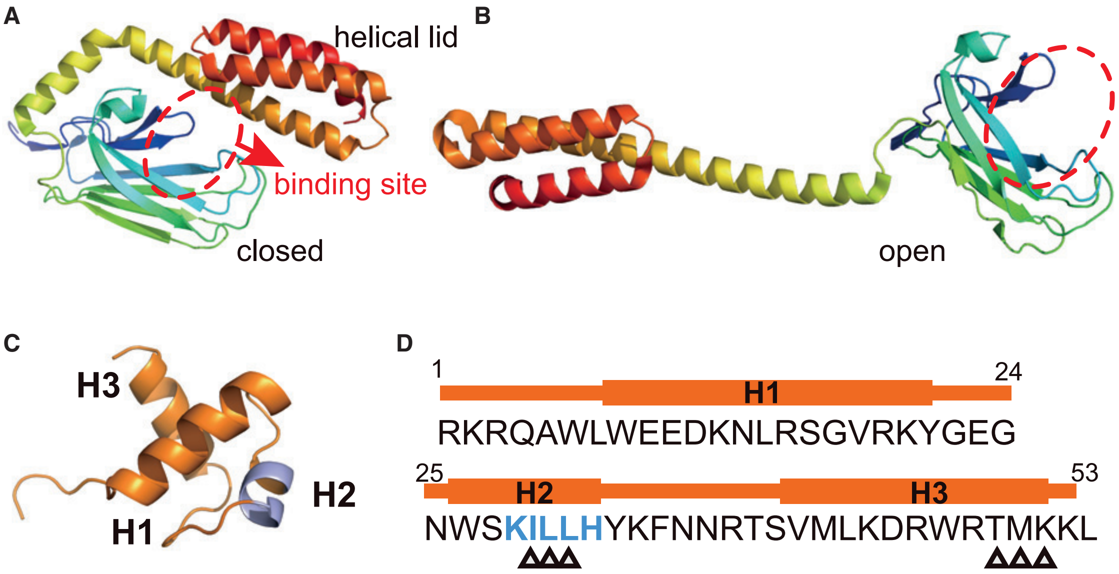

FIGURE 1 Three-dimensional structures of the chaperone and substrate. (*A* and *B*) Cartoon representations of the three-dimensional structures of the substrate binding domain of the bacteria Hsp70 (DnaK) at closed (*A*) and open (*B*) conformations (20,21). The substrate binding site is labeled with a red ellipse. (*C*) Cartoon representation of the native structure of the substrate hTRF1 (22). The native contacts are mainly formed between helices H1 and H2 and between helices H1 and H3. The residues contributing to the chaperone binding are marked by blue. (*D*) Amino acid sequence of substrate hTRF1, with the chaperone binding site being colored blue. The six residues with non-native contacting interactions are labeled by triangles. To see this figure in color, go online.

unclear, despite their biological significance. There are several classes of molecular chaperones involved in the protein homeostasis, among which the Hsp70 chaperones (e.g., DnaK in *Escherichia coli*) and Hsp60 (e.g., GroEL in *E. coli*) have been most exhaustively characterized. Early studies mainly focused on the GroEL chaperonin, based on which three distinct scenarios have been proposed to explain the working mechanisms of chaperones in protein folding (10–13). The most classic scenario is the Anfinsen cage model (14), whereby the chaperones prevent the nonproductive intermolecule aggregation in a passive way by providing an isolated environment or by protecting the exposed hydrophobic patches of substrate proteins (also described as holdase). The second scenario is the iterative annealing model or kinetic proofreading model (10–12,15–18), in which the chaperone actively converts the kinetically trapped misfolded conformations of substrate into unfolded conformation by acting as an unfoldase, allowing substrate proteins to refold. In addition to the above two scenarios, it has been showed that the confinement effect of the GroEL chaperonin cage tends to speed up folding by reducing the conformational entropy of the unfolded state, which suggests that chaperones can also facilitate the folding process as a foldase (19).

In recent years, Hsp70-mediated folding has attracted much attention. Different from the GroEL chaperonin, Hsp70 is a monomeric chaperone composed of two domains, i.e., the N-terminal nucleotide binding domain and the C-terminal substrate binding domain (SBD) (Fig. 1). The nucleotide binding states of the nucleotide binding domain allosterically regulate the conformations of the SBD (5). Upon ATP binding, Hsp70 adopts an open conformation with the hydrophobic substrate binding site exposed, allowing the binding or releasing of substrate proteins, whereas in the ADP or nucleotide-free state, the α-helical lid of the SBD closes, which leads to higher affinity of unfolded conformations and disruption of non-native hydrophobic contacts in misfolded conformations. By repeated allosteric cycles between the closed and open conformations driven by ATP binding and hydrolysis, the Hsp70 chaperone can either protect unfolded conformations or convert the misfolded substrate to unfolded conformations, which therefore contributes to increased fractions of productive folding (8,23,24). Such a functioning scenario is consistent with the holdase and unfoldase mechanisms revealed in the studies of the GroEL chaperonin. However, because of the lack of a cage architecture, whether Hsp70 can also act as a foldase to directly assist the folding process remains an open question. Recent experimental studies showed that Hsp70 in the ADP state can reduce the long-range contacts of the bound unfolded substrate, the human telomere repeat binding factor (hTRF1), which may therefore lead to biased folding pathways (25). Whether Hsp70 can continuously modulate the folding energy landscape and therefore facilitate the folding by directly assisting the subsequent folding procedure after the closed-to-open conformational change is still unclear. Experimentally addressing this question is extremely challenging because it is difficult to characterize the structure and dynamics of the substrate protein when it is continuously bound to the chaperone at the open

conformation. Molecular simulations provide an alternative way to elucidate the above question because of their ability to achieve unprecedented temporal and spatial resolutions. Such methods have been successfully used in investigating the functioning mechanisms of GroEL-mediated protein folding in previous studies (11,26–32). The typical timescales of the steps involved in the Hsp70 operational cycle are larger than millisecond. For example, the folding timescale of the substrate hTRF1 can be as long as ~3.5 ms (33), whereas the closed-to-open conformational change of Hsp70 occurs at the timescale of seconds (9). Such a long timescale is far beyond the accessibility of atomistic molecular simulations, and coarse-grained simulations are needed. In this work, by constructing a protein folding model with a rugged energy landscape, we performed coarse-grained molecular dynamics simulations for the Hsp70-mediated folding of hTRF1 (Fig. 1, *C* and *D*; (22)) and another two substrate proteins (SH3 and RNase H) (34,35). The results showed that the presence of the Hsp70 chaperone with an open conformation alters the folding pathways of the substrates by remodeling its energy landscape such that the folding dominantly follows the smooth pathway with reduced probability of misfolding. Particularly, we showed that altering the folding pathways requires the involvement of direct Hsp70-substrate interactions during the folding process. The results of this work are in line with previous experimental observations (25) and suggest a crucial role of the Hsp70 chaperone as a foldase during its functional actions.

MATERIALS AND METHODS

Protein model and energy functions

We used a coarse-grained model to simulate the folding and conformational motions of the substrate proteins and the Hsp70 chaperone, in which each amino acid is represented by one spherical bead locating at its C_α position. The intramolecule interactions of the substrate proteins were described by the following energy function:

$$V\left(R \mid R^0\right) = V_{\mathrm{loc}}(R) + V_{\mathrm{SB}}\left(R \mid R^0\right) + V_{\mathrm{KH}}(R) + V_{\mathrm{ele}}(R) \quad (1)$$

In the above formula, $V_{\mathrm{loc}}(R)$ represents the generic potentials for the angle (θ) and dihedral angle (ϕ) terms and is given by $V_{\mathrm{loc}}(R) = \sum_I V_a^I(\theta) + \sum_I V_{\mathrm{dih}}^I(\phi)$, with $V_a^I(\theta) = -k_B T \ln(P(\theta)/\sin(\theta))$ and $V_{\mathrm{dih}}^I(\phi) = -k_B T \ln(P(\phi))$. Here, R represents the coordinates of the coarse-grained beads in a given protein structure, and the index I runs over all the bond angles and dihedral angles. $P(\theta)$ and $P(\phi)$ are the distributions of the angles and dihedral angles formed by a certain combination of amino acids and were extracted by statistical survey of the coiled library (36). This generic local potential was developed in (36) and has been implemented in the software CafeMol used in the molecular dynamics (MD) simulations of this work (37). $V_{\mathrm{SB}}(R|R^0)$ corresponds to the structured-based term (38,39), which shapes up a funneled energy landscape driving the folding of the proteins to their native structures (38,40,41). Such structure-based models showed great success in describing many aspects of protein folding in previous studies (38,41,42). The coordinates of the reference native structures (R^0) were taken from the Protein Data Bank with the entry numbers PDB: 1BA5, 2A36, and 1F21 for hTRF1, SH3, and RNase H, respectively (21,22,43). The atomic-interaction-based coarse-grained model (AICG2+) developed in our previous work was used for the first two terms, i.e., $V^{AICG}(R|R^0) = V_{\mathrm{loc}}(R) + V_{\mathrm{SB}}(R|R^0)$, which is a structure-based potential with the interactions optimized using the multiscale strategy (44–47). The overall strengths of the structure-based interactions in the AICG2+ were controlled by a scaling factor λ_{SB} in AICG2+ (37,46), which were optimized to reproduce the experimentally observed stabilities of the studied proteins and were listed in Table S1. More details of the AICG2+ energy function can be found in (46). For the residue pairs without forming direct contacts in the native structures, we also applied a nonspecific knowledge-based statistical potential $V_{\mathrm{KH}}(R)$, and the energy function proposed by Kim and Hummer was used (48). For the charged residue pairs, the electrostatic interaction term $V_{\mathrm{ele}}(R)$ was applied, which is given by the Debye-Hückel formula (49).

The above AICG2+ energy function corresponds to a minimally frustrated landscape (40). Because the molecular chaperones mostly act on proteins with rugged energy landscapes, which encounter the risk of misfolding, additional frustration needs to be included in the energy function of the substrate. Although the generic statistical potential ($V_{\mathrm{KH}}(R)$) and electrostatic potential ($V_{\mathrm{ele}}(R)$) in Eq. 1 can make the energy landscape more rugged, the resulting frustration is not strong enough to create a misfolded state, which is often a feature of the substrate proteins of chaperones. Therefore, we also added a non-native contacting interaction term to the above energy function to shape up a rugged energy landscape so that the population of the long-lived misfolded state is significant. For the three proteins studied in this work, the residues that were bound with (and therefore protected by) the Hsp70 chaperone were known in experiments (33–35). Therefore, we introduced the non-native contacting interactions specifically between these residues and some other residues spatially distant in the native structure to produce a long-lived misfolded state. For example, as shown in the NMR structure of hTRF1 (Fig. 1 *C*), helix 1 (H1) forms native contacts with H2 and H3, whereas contacts between H2 and H3 are rare. To discuss the role of the Hsp70 chaperone in the folding of hTRF1, we introduced additional contacting interactions between H2 and H3, with which the folding of the substrate hTRF1 may involve a kinetic trap that features incorrect packing between H2 and H3. The residue pairs involved in the non-native contacting interactions were listed in Table S2 for all three substrate proteins, and the energy function of the non-native contacting interactions were given by the following Lennard-Jones potential:

$$V_{\mathrm{nnat}}\left(r_{ij}\right) = \varepsilon_{\mathrm{nnat}}\left[5.0\left(\frac{\sigma}{r_{ij}}\right)^{12} - 6.0\left(\frac{\sigma}{r_{ij}}\right)^{10}\right], \quad (2)$$

with r_{ij} being the distance between the two contacting residues and $\sigma = 5.0$ Å being the position of the energy minimum. The parameter $\varepsilon_{\mathrm{nnat}}$ is the strength of the non-native contacting interactions characterizing the frustration extent of the energy function. In the following discussions, we used a wide range of values for the parameter $\varepsilon_{\mathrm{nnat}}$ to test the parameter sensitivity. We also performed simulations with different number of residue pairs involved in the non-native contacting interactions (Table S3).

According to previous works (20,50,51), Hsp70 adopts closed and open conformations in the ADP and ATP states, respectively, demonstrating the ATP hydrolysis-mediated allosteric feature. To reasonably describe such an allosteric feature of Hsp70, we designed an energy function with double-basin topography for the intramolecule interactions of the Hsp70 chaperone following the scheme proposed by Okazaki and co-workers (52), which was given by

$$V_s^{\mathrm{Hsp}}(R) = \frac{V^{\mathrm{AICG}}\left(R \mid R_0^T\right) + V^{\mathrm{AICG}}\left(R \mid |_0^D\right) + \Delta V_s}{2}$$

$$-\sqrt{\left[\frac{V^{\mathrm{AICG}}\left(R \mid R_0^T\right) - V^{\mathrm{AICG}}\left(R \mid R_0^D\right) - \Delta V_s}{2}\right]^2 + \Delta_s^2}, \quad (3)$$

where the subscript s = T, D and represents the ATP state and ADP state. The coordinates of the two reference structures R_0^T and R_0^D were taken from the Protein Data Bank with entry numbers PDB: 4B9Q and 4EZW (21,43), corresponding to the closed structure and open structure, respectively. The parameter set ΔV_s and Δ_s were chosen so that Hsp70 dominantly adopts a closed (open) conformation in the ADP (ATP) state following the experimental restraints (Table S4; (50)). Although this model cannot be directly used to model the nucleotide exchange process, the conversion from the ADP state to the ATP state of Hsp70 can be effectively described by the switching of parameters in the energy function from the ADP state to the ATP state.

The interactions between the chaperone and substrate are given by the following hydrophobicity-dependent energy function proposed in a previous work (37,53):

$$V_{\mathrm{CS}}(R) = c_{\mathrm{HP}}\sum_i \varepsilon^i_{\mathrm{HP}} S^i_{\mathrm{HP}}(R) + \sum_{i,j} \varepsilon_{\mathrm{exv}}\left(\sigma_{ij}/r_{ij}\right)^{12} \quad (4)$$

In the first term of the above formula, the index i runs overall all the residues of the Hsp70 and the residues in the chaperone binding sites of the substrate proteins. c_{HP} controls the overall strength of the hydrophobic interactions. The default value of c_{HP} was set as 5.0, but we also performed the simulations with different c_{HP}-values. $\varepsilon^i_{\mathrm{HP}}$ corresponds to the parameter set describing the hydrophobicity scores of the residues in the binding sites, which were listed in Table S5. $S^i_{\mathrm{HP}}(R)$ describes the "buriedness" of the residue i, which depends on the packing density around the residue i. More details of the energy function and parameters can be found in (37,53). The second term describes the excluded volume interactions, with $\varepsilon_{\mathrm{exv}} =$ 0.2 kcal/mol and $\sigma_{ij} = (\sigma_i + \sigma_j)/2$. Here, the index i and j run over all the residues of the substrate and the chaperone, respectively. σ_i is the radius of residue i (Table S6; (48)).

Molecular simulations

Molecular simulations were conducted by using the software CafeMol3.0 (37). The simulation temperature was set as 300 K and controlled by Langevin dynamics. The friction coefficient was set as $0.08\tau^{-1}$, with $\tau \sim$49 fs being the time unit used in the CafeMol (37). In the simulations, only the substrate binding domain of Hsp70 was included. The functioning cycle of Hsp70 features allosteric motions driven by ATP binding and hydrolysis. In this work, we did not consider ATP binding and hydrolysis explicitly because of the computational complexity. Instead, the allosteric cycle was modeled as the conformational switching between the open and closed states realized by switching the energy functions of Hsp70 from the ADP state to the ATP state, which triggers the closed-to-open conformational change.

Before folding simulations, we prepared two unfolded structural ensembles of the substrate hTRF1 by using thermal denaturation and Hsp70 actions, respectively, by which we can investigate whether the difference of the initial structure ensembles can alter the folding pathways. The unfolded structures of the thermal denaturation ensemble were sampled by equilibrium simulations at high temperature for 5×10^8 MD steps, with the MD time step of 0.4τ such that the secondary structure elements and long-range contacts have been mostly disrupted. We note that the timescale used in coarse-grained model often corresponds to a much longer realistic timescale as discussed in (47), although precise timescale mapping is always difficult. To ensure that all the secondary structures are disrupted, we used a sufficiently high temperature (800 K) in the thermal denaturation simulations. In comparison, the structures of the Hsp70-induced unfolded ensemble were sampled by equilibrium simulations with the substrate hTRF1 bound to the closed conformation of Hsp70 for 2×10^8 MD steps. The simulations were conducted at 300 K so that the secondary structure elements remained well formed, whereas the long-range contacts were disrupted because of the strong binding of the Hsp70 chaperone. Therefore, the two unfolded ensembles have different secondary structure contents. Folding simulations starting from these two different unfolded ensembles can be used to investigate the effect of the initial unfolded structures on the folding kinetics.

In this work, we performed three different kinds of folding simulations, including 1) T-unfolded simulations, 2) Hsp70-unfolded simulations, and 3) Hsp70-mediated simulations. In the T-unfolded simulations, the folding starts from the thermally unfolded ensemble and proceeds spontaneously without the involvement of Hsp70 chaperone, whereas in the Hsp70-unfolded simulations, the folding starts from the unfolded ensemble prepared by Hsp70 action and proceeds spontaneously without the involvement of Hsp70 chaperone. In the Hsp70-mediated simulations, the folding starts from the unfolded ensemble prepared by Hsp70 action. Then Hsp70 switches to the open conformation, and the folding proceeds with the presence of the Hsp70 chaperone.

Data analysis and reaction coordinates

To characterize the Hsp70-mediated folding of hTRF1, we introduced the reaction coordinates Q, Q_{12}, Q_{13}, N_{23}, and N_{mis}. The reaction coordinate Q describes the fraction of the formed native contacts in a given structure, which is defined by

$$Q = \frac{1}{N_{\mathrm{nat}}}\sum_{ij}\frac{1}{1+\exp\left[-\beta\left(\lambda - r_{ij}/r^0_{ij}\right)\right]}, \quad (5)$$

where N_{nat} is the number of native contacts in the native structure of hTRF1. The sum index runs over all the residue pairs forming the native contacts. r_{ij} and r^0_{ij} are the distances of the residue pair in a given snapshot and in the native structure, respectively. β and λ are two parameters with the values of 10.0 and 1.2, respectively. Similarly, Q_{12} and Q_{13} describe the fractions of the formed native contacts between H1 and H2 and between H1 and H3, respectively. N_{23} is the total number of formed contacts between H2 and H3 in a given structure, which is calculated as $N_{23} = \sum_{i\in H2, j\in H3} 1/[1 + \exp(-\beta(\lambda - r_{ij}/r^0))]$, with $r^0 = 6.5$ Å. Therefore, N_{23} not only includes the formed native contacts between H2 and H3 but also includes all the other residue pairs spatially close between H2 and H3 in a given structure. The N_{mis} is the number of formed contacts for the residue pairs with non-native contacting interactions given in Eq. 2. The two-dimensional free energy profiles were calculated by $F(RC_1, RC_2) = -\ln P(RC_1, RC_2)$, with the RC_1 and RC_2 being two reaction coordinates.

To characterize the folding pathways of the substrate hTRF1, we defined five conformational states, including the unfolded state (U), native state (N), misfolded intermediate (I_{23}), and two on-pathway intermediates (I_{12} and I_{13}). These states were defined by the conditions ($Q_{12} < 0.3$, $Q_{13} < 0.3$, and $N_{\mathrm{mis}} = 0$); ($Q \geq 0.9$); ($N_{\mathrm{mis}} \geq 1$); ($Q_{12} \geq 0.8$, $Q_{13} < 0.3$, $Q < 0.9$, and $N_{\mathrm{mis}} = 0$); and ($Q_{12} < 0.3$, $Q_{13} \geq 0.8$, $Q < 0.9$, and $N_{\mathrm{mis}} = 0$), respectively. The folding pathway was then represented by a sequence of the above states in a trajectory, with the loops being removed. The transition state ensemble was constructed by the structures in the transition path of the substrate folding events with $0.6 \leq Q \leq 0.8$. The molecular structures were visualized by the software VMD and PyMol (54,55). Q, N_{mis}, and the misfolded intermediate were defined similarly for the substrates SH3 and RNase H. The mean first passage time (MFPT) of the substrate folding was calculated based on the folding trajectories by a maximal likelihood estimation.

RESULTS

Spontaneous folding and kinetic trap

To characterize the conformations accessible by hTRF1 with a rugged energy landscape, we conducted molecular

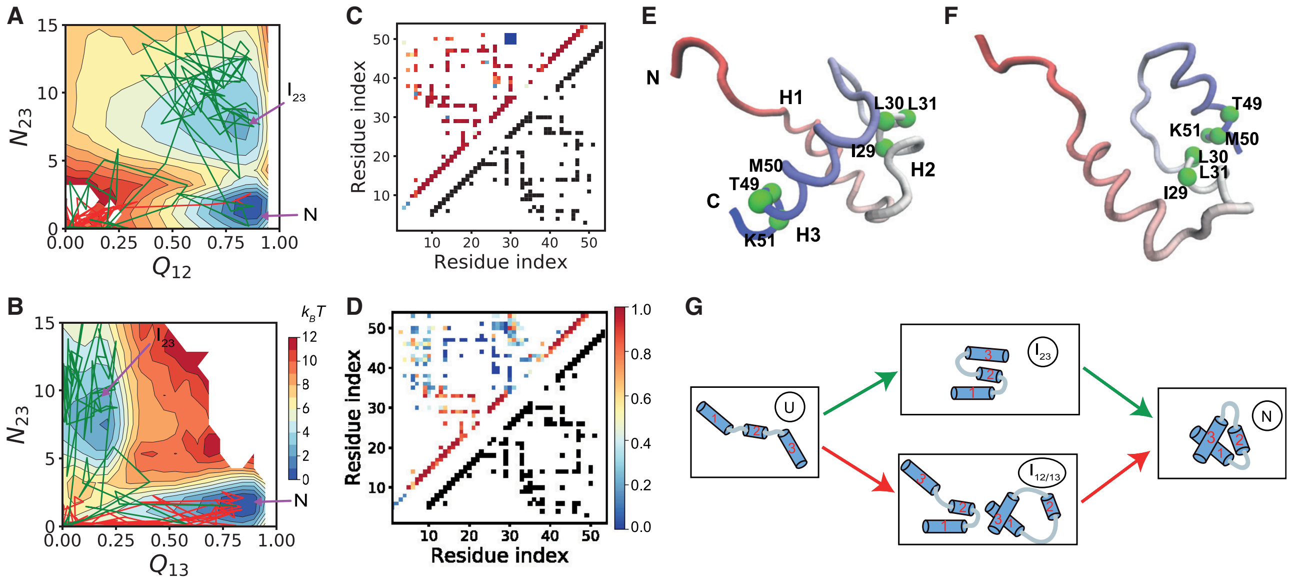

FIGURE 2 Kinetic trap and folding pathways in the spontaneous substrate folding. (*A* and *B*) Two-dimensional free energy profiles of hTRF1 along the reaction coordinates (Q_{12}, N_{23}) (*A*) and (Q_{13}, N_{23}) (*B*). The native state ("*N*") and misfolded state ("I_{23}") were labeled. (*C* and *D*) Probabilities of the contact formation for the sampled conformations in the native state (*C*) and misfolded state (*D*). The results for residue pairs not forming native contacts are also shown when the probabilities exceed 0.1. For reference, all the native contacts are shown by the black squares in the lower-right part. (*E* and *F*) Representative structures of hTRF1 in the native (*E*) and misfolded (*F*) states. The green spheres represent the residues with non-native contacting interactions. (*G*) Cartoon schematic of the smooth pathway (*red*) and frustrated pathway (*green*) for hTRF1 folding. Note that the transition from the state I_{23} to the state N may involve other states, which are omitted in the scheme for clarity. To see this figure in color, go online.

simulations for the isolated substrate hTRF1 (without the Hsp70 chaperone) at 300 K. The two-dimensional free energy profiles based on 20 independent simulations with the length of 5×10^8 MD steps show two major free energy basins (Fig. 2, *A* and *B*), which were labeled as "I_{23}" and "*N*," respectively. To more clearly illustrate the structure features of the two major conformational states, we calculated the contact probability maps based on the sampled structures (Fig. 2, *C* and *D*). For the conformations locating at the basin "*N*," the native contacts between the helices H1 and H2 and between the helices H1 and H3 are largely formed, whereas the contacts between H2 and H3 are mostly lacking (Fig. 2 *C*), which corresponds to the native-like state (Fig. 2 *E*). In comparison, for the conformations locating at the basin "I_{23}," a number of non-native contacts between H2 and H3 are formed, whereas the native contacts are disrupted to a large extent. Therefore, the state "I_{23}" corresponds to a kinetically trapped misfolded state that features the formation of non-native contacts (Fig. 2 *F*).

Because of the presence of the kinetic trap, the folding of the substrate protein can follow two major pathways, i.e., a smooth pathway and a frustrated pathway (Materials and methods). In the smooth pathway, the substrate protein folds to the native structure via the on-pathway intermediate I_{12} or I_{13} without involving the misfolded state (Fig. 2 *G*, *red arrow lines*). In the frustrated pathway, the folding involves the misfolded state I_{23} (Fig. 2 *G*, *green arrow lines*). The representative folding trajectories following the above two folding pathways were also plotted in the two-dimensional free energy profiles. For the frustrated pathway (*blue*), starting from the unfolded initial structure (U), the folding of the substrate protein was trapped into a misfolded state for a long time before it folds to the native structure, which therefore tends to slow down the folding kinetics. Such a frustrated folding model will be used to investigate the role of the Hsp70 chaperone on substrate folding in the subsequent discussions.

Protein folding mediated by Hsp70 chaperone

Next, we performed molecular simulations of hTRF1 folding along the rugged energy landscape with the presence of the Hsp70 chaperone. The operational cycle of Hsp70 involves substrate binding and unbinding, nucleotide exchange and ATP hydrolysis, and conformational changes, which are regulated by the binding of other co-chaperones such as Hsp40 and nucleotide exchange factors, and the related timescale of the cycle can be as long as seconds. Therefore, directly simulating the whole cycle is extremely difficult even with a coarse-grained model. Here, the allosteric cycle of Hsp70 was modeled by switching the energy functions from the ADP state to the ATP state, which triggers the closed-to open conformational change of Hsp70 (Table S4). Firstly, the substrate with extended form was put into the binding pocket of Hsp70. After that, a long equilibrium simulation of 2×10^8 MD steps was performed to sample the Hsp70-bound ensemble at closed conformation (Fig. 3 *A*, *left*). Such unfolded structures prepared by

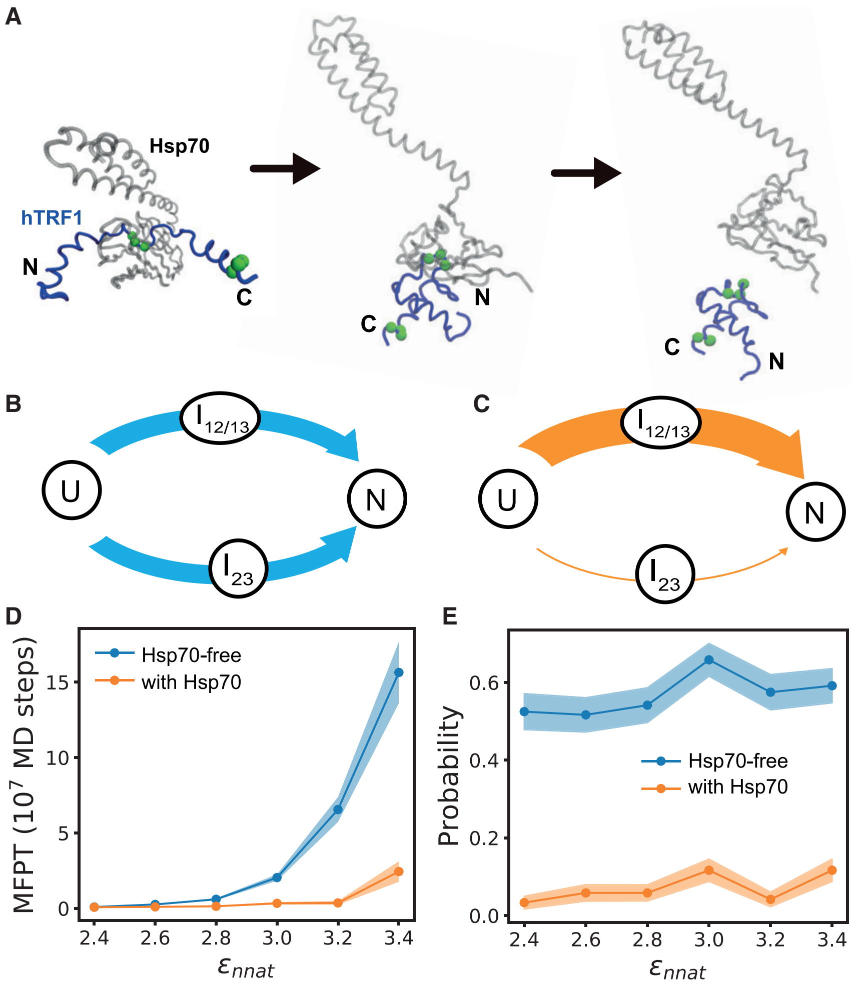

FIGURE 3 Hsp70-mediated folding of the hTRF1. (*A*) Cartoon representation of the chaperone-substrate system at three representative time snapshots during Hsp70-mediated folding. (*B* and *C*) Populations of the folding pathways without (*B*) and with (*C*) the presence of the Hsp70 chaperone calculated with $\varepsilon_{\mathrm{nnat}}$ of 2.6 kcal/mol. The line breadth represents the probabilities of the pathways. (*D* and *E*) Mean first passage time (MFPT) (*D*) and probabilities of the frustrated pathway (*E*) with (*orange*) and without (*blue*) Hsp70 for the simulations with different frustration extents. The standard errors are shown by shaded areas. To see this figure in color, go online.

Hsp70 actions constitute the unfolded ensemble and will be used as the initial structures for subsequent folding simulations. Then, Hsp70 was switched to the open conformation, and the substrate protein started to fold (Fig. 3 *A*, *middle*). Interestingly, in our simulations, the substrate protein can stay bound to Hsp70 for a long time; therefore, the folding occurs with the substrate continuously interacting with the chaperone. Once the substrate folded to the near-native structure ($Q > 0.8$), the Hsp70 chaperone was removed (Fig. 3 *A*, *right*).

To characterize the effect of Hsp70 on the folding kinetics, we performed 120 independent folding simulations with and without the presence of the Hsp70 chaperone at 300 K. The folding simulations without Hsp70 were initiated from the starting structures prepared by thermal denaturation. Therefore, the whole folding process, including the initial unfolded structure and the subsequent folding procedure, is not affected by the Hsp70 chaperone, whereas the folding with the Hsp70 chaperone was initiated from the starting structures prepared by the unfolding actions of Hsp70 described above. The difference between the results from the above two simulations can be used to represent the effect of Hsp70 on the substrate folding. Without the presence of Hsp70 (Hsp70-free), there are large fractions of folding pathways going through the misfolded state I_{23} (~50%, Fig. 3 *B*). In comparison, with the presence of the Hsp70 chaperone, the folding dominantly follows the smooth pathway, and the population of the frustrated pathway is minor (~6%, Fig. 3 *C*). Correspondingly, the MFPT of the folding was reduced by threefold. The above results clearly suggest that the Hsp70 chaperone with an open conformation is able to alter the folding pathways such that the frustrated pathway is much reduced, which leads to the acceleration of the overall folding kinetics.

In the above discussions, the default value for the strength of the non-native contacting interactions ($\varepsilon_{\mathrm{nnat}} = 2.6$ kcal/mol) was used (Materials and methods). This parameter controls the frustration extent of the folding landscape. Because different substrate proteins may involve different extents of folding frustration, it is interesting to investigate how the effect of the Hsp70 chaperone depends on the frustration extent. We therefore performed similar simulations with a

wide range of $\varepsilon_{\rm nnat}$-values. As expected, for the Hsp70-free simulations, the folding time increases drastically with an increase of frustration (Fig. 3 *D*, *blue*). For example, with the $\varepsilon_{\rm nnat}$-value of 2.4 kcal/mol, the MFPT is 0.095×10^7 MD steps. When the $\varepsilon_{\rm nnat}$ increases to 3.4 kcal/mol, the MFPT becomes 15.6×10^7 MD steps, which amounts to a 164-fold increment in the folding time. In comparison, the MFPT only increases slightly for the Hsp70-chaperone-mediated folding (Fig. 3 *D*, *orange*). As a consequence, the effect of the Hsp70 chaperone becomes much more significant for folding with larger frustrations. The population of the folding pathways are not changed by the values of the $\varepsilon_{\rm nnat}$ (Fig. 3 *E*). Therefore, the increase of the MFPT in the Hsp70-free simulations mainly arises from the elongated time for escaping from the misfolded state.

The above results demonstrated that the effect of Hsp70 in the substrate folding becomes more significant when the extent of frustration increases. In the above discussions, we introduced the non-native interactions for nine pairs of residues (Table S2), by which a value of $\varepsilon_{\rm nnat}$ larger than 2.6 kcal/mol is needed to produce sufficient frustration, so that the effect of Hsp70 can be observed (Fig. 3 *D*). We also performed simulations with 25 (2) residue pairs being involved in the non-native contacting interactions, and the threshold value of $\varepsilon_{\rm nnat}$ becomes much smaller (larger) (Figs. S1 and S2). The main results are insensitive to the setup of the non-native contacting interactions (therefore, the $\varepsilon_{\rm nnat}$-values). For the hydrophobic interactions between Hsp70 and substrates, we used the parameter $c_{\rm HP}$ to control the interaction strengths. In this work, a default value of $c_{\rm HP} = 5.0$ was used. The resulting average strength per contact is $\sim$1.0 kcal/mol. We also performed simulations with different values of $c_{\rm HP}$, and the main results are similar (Figs. S3 and S4).

It is interesting to investigate the molecular events occurring during the conformational switching. Starting from the closed conformation with the substrate bound, we collected the transition path trajectories from 120 independent simulations, which are terminated once they arrive at the open conformation for the first time. The results showed that during the closed-to-open conformational switching, the intramolecule contacts of the substrate do not change (Fig. S5), which suggests that the opening of the Hsp70 conformation cannot perform conformational work to the substrate, consist with the observation in a previous NMR measurement (33). As expected, the opening of Hsp70 is accompanied by the decreasing of the chaperone-substrate contacts and the widening of the substrate binding groove (Fig. S5), which leads to increased freedom for the substrate to sample the structures with more native contacts.

Hsp70 chaperone facilitates substrate folding by remodeling the energy landscape

In the above discussions, the Hsp70-free simulations were initiated from the unfolded structures prepared by thermal unfolding. To better understand the role of Hsp70 in substrate folding, we also performed Hsp70-free simulations with the unfolded structures prepared by Hsp70 actions (see Materials and methods for the preparation of unfolded ensembles). Although the three helices are better folded in the unfolded ensemble prepared by Hsp70 actions (Fig. 4 *A*) compared with that from thermal unfolding (Fig. 4 *B*), both ensembles are lacking long-range contacts, and the non-native contacting interactions were fully eliminated. The results showed that the folding time and populations of the Hsp70-free folding pathways starting from these two unfolded ensembles are comparable. Such results may suggest that modulation of long-range tertiary interactions of the unfolded structures plays a crucial role in modifying the folding kinetics by Hsp70, which is consistent with the experimental data shown in (25).

As discussed above, the substrate protein hTRF1 remains bound to Hsp70 until it folds to the near-native structure ($Q \geq 0.8$) in the Hsp70-mediated folding. Therefore, the effect of Hsp70 on the substrate folding can be attributed to the direct chaperone-substrate interactions. The two-dimensional free energy profiles constructed based on the sampled conformations with the substrate bound to the closed (Fig. 4, *E* and *F*) and open (Fig. 4, *G* and *H*) states of Hsp70 are dramatically distinct from that of the free substrate (Fig. 2 *A*). Particularly, at the open conformation (Fig. 4, *G* and *H*), the conformations corresponding to the misfolded state can hardly be sampled because of the direct interactions between the chaperone and substrate. Such results demonstrated that direct chaperone-substrate interactions tend to remodel the substrate energy landscape even after the closed-to-open conformational change of the Hsp70 chaperone, which then leads to biased folding pathways. To further test this mechanism, we then conducted folding simulations with the substrate continuously bound to Hsp70 at the early stage of the folding. All these folding simulations start from the same unfolded structural ensemble prepared by an Hsp70 action. As control, "Hsp70-unfolded" (the second low in Fig. 4, *C* and *D*) represents the simulations for which the folding starts from the same unfolded structural ensemble but without direct Hsp70-substrate interactions at the open state. When the Q-value exceeds a certain threshold $Q_{\rm th}$, the substrate releases from the chaperone. Larger values of $Q_{\rm th}$ mean that the substrate remains bound to the open state of the chaperone for longer time. In Fig. 4, *C* and *D*, we show the results of the simulations with $Q_{\rm th} = 0.4$, 0.6, and 0.8 at different $\varepsilon_{\rm nnat}$ and compared to the result without direct Hsp70-substrate interactions during the folding process (Hsp70-unfolded). One can see that with the increase of $Q_{\rm th}$, the folding of the substrate gets faster and the population of the frustrated pathway becomes smaller at a wide range of the frustration extents. The effect of $Q_{\rm th}$ on the folding time is more significant for the simulations with larger frustrations. These results clearly demonstrated that the Hsp70 chaperone not only modifies the structural ensemble of the unfolded

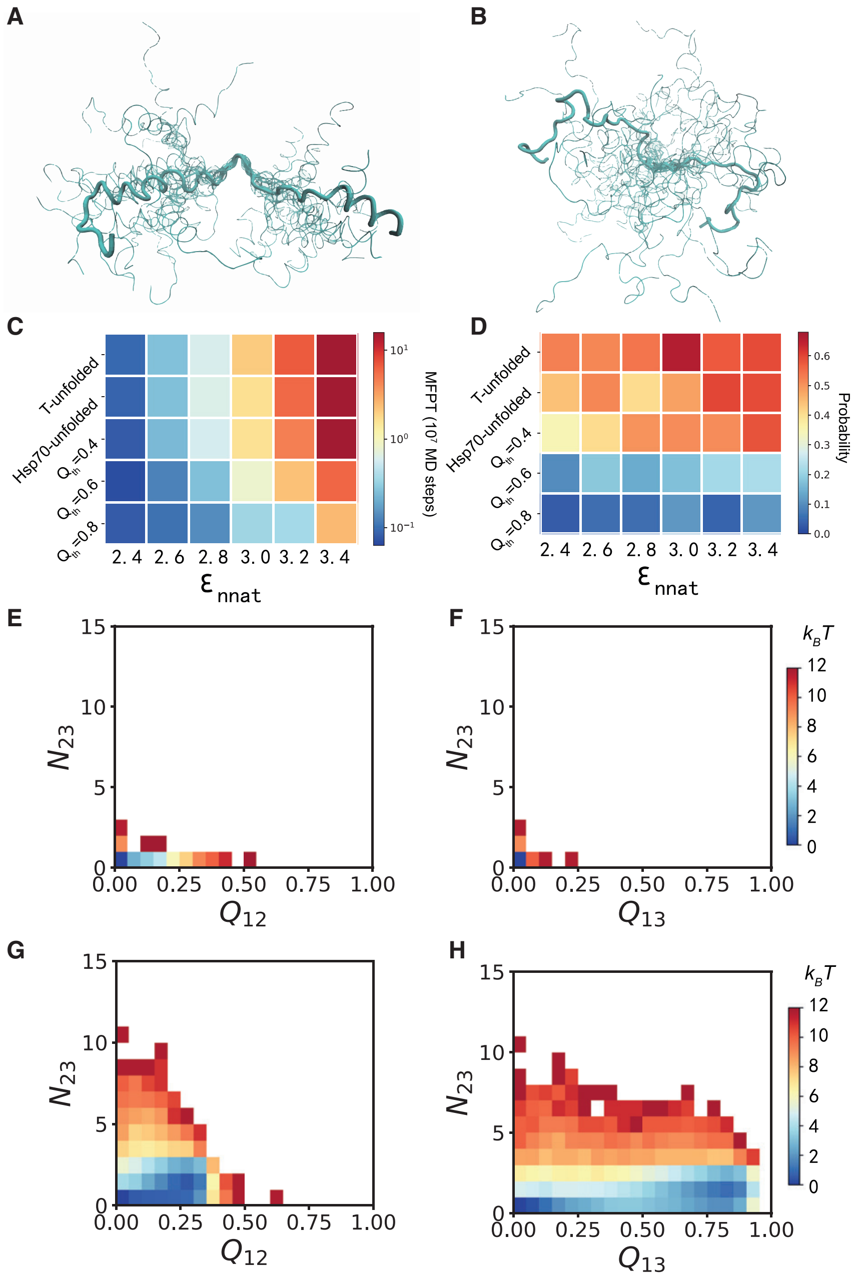

FIGURE 4 Role of Hsp70 in the substrate protein folding. (*A* and *B*) Unfolded conformations prepared by Hsp70 actions (*A*) and thermal unfolding (*B*). 20 structures are shown, with the residue segment 28–32 being aligned. (*C* and *D*) MFPT of substrate folding (*C*) and populations of the frustrated folding pathway (*D*) calculated based on different simulation strategies and frustration extents. T-unfolded: spontaneous folding starting from the initial structures prepared by thermal denaturation; Hsp70-unfolded: spontaneous folding starting from the initial structures prepared by unfolding action of Hsp70; Q_{th} = 0.4, 0.6, and 0.8: Hsp70-mediated folding with the chaperone being removed when the Q score exceeds 0.4, 0.6, and 0.8, respectively. (*E–H*) Two-dimensional free energy profiles of the substrate hTRF1 bound at the closed (*E* and *F*) and open (*G* and *H*) conformations of the Hsp70 chaperone. To see this figure in color, go online.

state when bound at the closed conformation of Hsp70 but also can be involved in the whole folding procedure even after the closed-to-open conformational switching. It alters the folding pathways and accelerate the folding events mainly by direct chaperone-substrate interactions during folding, which implies a foldase scenario of Hsp70-mediated folding. In addition, the substrate protein can access a much larger range of the conformational space when bound at the open conformation of Hsp70 compared to that at the closed conformation (Fig. 4, *E–H*).

To investigate whether the above-discussed role of Hsp70 on the substrate folding also applies to other substrate proteins, we conducted similar simulations for another two proteins, i.e., SH3 and RNase H, which are two substrate proteins of Hsp70 and have been studied in previous experimental works (34,35). Different from the all-α protein hTFR1 discussed above, these two proteins have the all-β and $\alpha + \beta$ structural classes, respectively. Similar to the results for hTRF1, one can see that with the increase of Q_{th}, the folding of the substrate gets faster if the introduced frustration is significant (Fig. 5, *A* and *C*). In addition, the population of the frustrated pathway becomes smaller with the increase of Q_{th} (Fig. 5, *B* and *D*). Such results suggest that directing the folding process of the substrate by direct

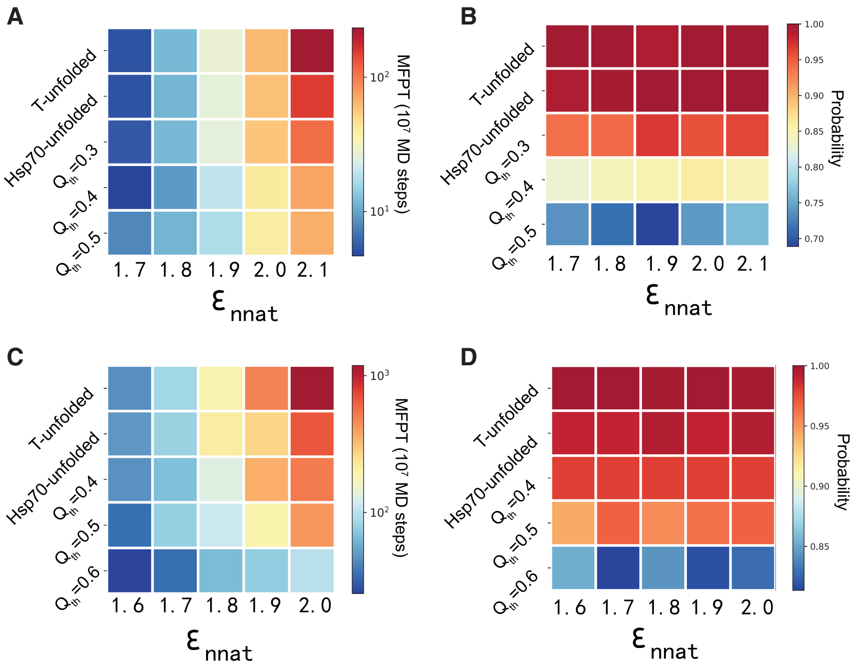

FIGURE 5 Folding kinetics for the substrate proteins SH3 and RNase H. (*A* and *B*) MFPT (*A*) and populations of the frustrated folding pathway (*B*) calculated based on different simulation strategies and frustration extents for the substrate protein SH3. (*C* and *D*) MFPT (*C*) and populations of the frustrated folding pathway (*D*) calculated based on different simulation strategies and frustration extents for the substrate protein RNase H. The folding pathway was considered as frustrated if the folding occured via the misfolded intermediate state. To see this figure in color, go online.

Hsp70-substrate interaction may be a generic working mechanism for the Hsp70 chaperone.

Modulation of transition state ensemble

The above results showed that involvement of Hsp70 modifies the folding kinetics by eliminating the frustrated pathways. In fact, because of the energy landscape remodeling, the relative populations of the two smooth subpathways via the intermediates I_{12} and I_{13}, respectively, were also altered by Hsp70 binding. As illustrated by the structural features of the transition state ensembles (Materials and methods), without the presence of Hsp70, the native contacts of the H1-H2 interface are formed to a large extent (Fig. 6), whereas those of the H1-H3 interface are mostly absent. In comparison, for Hsp70-mediated folding, formation of the H1-H2 contacts becomes less probable, and the H1-H3 contacts can form to some extent. Such results suggest that binding of Hsp70 not only modifies the relative populations of the smooth and frustrated folding pathways but also leads to kinetic repartitioning between the two smooth subpathways. Similar results can also be observed by analyzing the contact maps of the transition state ensembles (see Fig. S6) and the folding orders of different parts of the hTRF1 (see Fig. S7).

DISCUSSION

The molecular mechanisms of Hsp70-mediated protein folding have been the focus of recent efforts in protein folding studies. Although the canonical holdase and unfoldase mechanisms have been well characterized for Hsp70 (24,56), whether it can also function as a foldase to directly assist the substrate folding is still in dispute (23,24,57,58), as it is challenging in experiment to precisely characterize the folding kinetics with the presence of direct chaperone-substrate interactions.

The molecular simulations conducted in this work clearly demonstrated the foldase mechanism of the Hsp70 functional action (Fig. 7). We showed that after Hsp70 converts to the open conformation during its functional cycle, the substrate hTRF1 is not immediately released from the chaperone. Instead, the substrate starts to fold when it remains bound with the chaperone. Because the hydrophobic residues of the substrate protein (i.e., Ile29, Leu30, and Leu31 of the hTRF1), which are prone to form non-native contacts, are protected by Hsp70 binding, the tertiary interactions of the substrate favor correct folding and are less inclined to misfolded structures. Therefore, the interactions arising from chaperone binding are able to remodel the intrinsic energy landscape of the substrate folding, leading to alteration of the folding pathways. Without the assistance of the Hsp70 chaperone, more than half of the folding events go through the frustrated pathway, which features an encounter with the misfolded state. In comparison, the majority of the folding events follow the smooth pathway when the chaperone is involved in the folding process. It is worth noting that altering the folding pathways by direct Hsp70-substrate interactions during the folding process does not cost free energy from ATP hydrolysis. However, because the Hsp70-mediated substrate folding is one part of the whole Hsp70 operational cycle, the ATP hydrolysis free energy is needed to drive the Hsp70 cycle, which makes the folding step occur repeatedly. According to previous studies (20,43,50,51), the

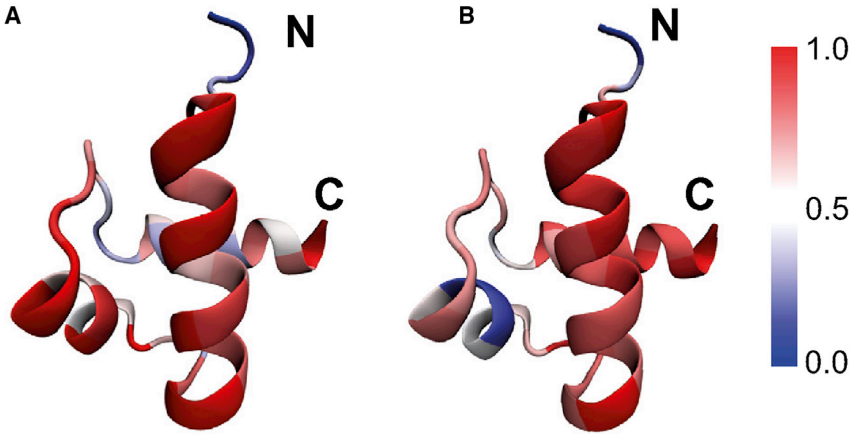

FIGURE 6 Color maps showing the probabilities of native contact formations in the transition state ensembles without (*A*) and with (*B*) the presence of the Hsp70 chaperone. To see this figure in color, go online.

hydrolysis of ATP triggers the open-to-closed conformational change of the substrate binding domain, which is one of the key steps of the Hsp70 operational cycle.

Interestingly, in a recent experimental work (25), Sekhar et al. measured the structural features of the substrate hTRF1 with NMR at the free form and bound at the closed state of the Hsp70 by using the ^{1}H-based chemical exchange saturation transfer (^{1}H CEST), with which the otherwise invisible state can be measured. They showed that the binding of Hsp70 in the ADP state (closed conformation) can significantly reduce the residual long-range (potentially nonnative) interactions that are encountered in the free form of the substrate at water solvent. The structural feature of the Hsp70-bound substrate is similar to that of 4 M urea denatured substrate concerning the residual long-range interactions. Based on these observations, the authors proposed that Hsp70 can modify the folding pathways of substrate proteins by removing the long-range interactions. The elimination of the non-native contacts due to the binding of Hsp70 observed in the current MD simulations is consistent with the above experimental results. Here, the MD simulations showed that the Hsp70-substrate interactions continuously play a role in the whole folding process even after the closed-to-open conformational change of Hsp70, therefore demonstrating a foldase mechanism.

The crucial role of the direct chaperone-substrate interactions during substrate folding was also demonstrated by the molecular simulations of hTRF1 folding starting from the unfolded structures prepared by Hsp70 action (rich in secondary structures), but with the chaperone being removed during folding. The resulted folding kinetics is similar to that of the spontaneous folding starting from the thermally denatured structures (lacking in secondary structures), which suggests that continuously modulating the long-ranged tertiary interactions during folding are more crucial than altering the secondary structure contents of the initial unfolded structures for the Hsp70-chaperone-mediated substrate folding, once the long-range non-native contacts in the initial unfolded structures were eliminated.

The above-discussed energy landscape remodeling mechanism of Hsp70-mediated protein folding, as shown by the involvement of direct chaperone-substrate interactions during folding, can be supported by a number of previous experimental observations (59–61). For example, based on electron paramagnetic resonance spectroscopy measurement, Schlecht and co-workers showed that the substrate proteins bound with the Hsp70 chaperone can adopt a wide spectrum of conformations, including not only the conformations with extended structures but also the conformations with substantial tertiary structures (59), which implies the feasibility of substrate folding while it remains bound with the chaperone. A single-molecule experiment by Mashaghi et al. also revealed the conformational plasticity of the substrate binding site and its ability to accommodate near-native substrate structures (60). In addition, recent experimental studies showed that the Hsp70 chaperones bound to the substrate protein release in an asynchronous mechanism (61), which suggests that some chaperones may stay bound with the substrate at different stages of the folding and therefore may modulate the energy landscape and folding pathways. The direct chaperone-substrate interactions during substrate folding have also been suggested for other ATP dependent and independent chaperones (62–65). Therefore, remodeling the intrinsic energy landscape of the substrate proteins by continuous chaperone-substrate interactions during folding can be a common mechanism adopted by various of chaperones. It is worth noting that the energy landscape remodeling mechanism discussed in this work, which is in correspondence to the foldase scenario, is not exclusive of the canonical holdase and unfoldase scenarios. On the contrary, they represent different aspects for the functions of the chaperones in assisting the folding of substrate proteins. For example, Hsp70 binding may lead to unfolding of a misfolded substrate (i.e., acting as an unfoldase), from which subsequent folding can occur. Although directly simulating such unfoldase action is beyond the capability of this model because of the involvement of co-chaperone

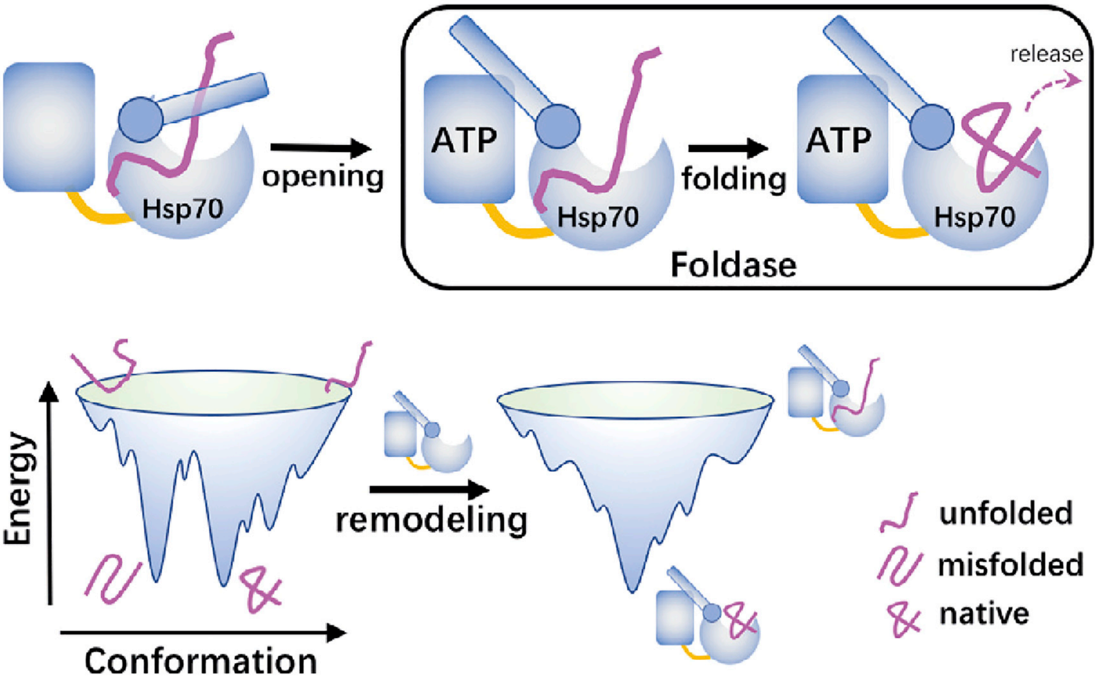

FIGURE 7 Cartoon diagram showing the foldase mechanism of the Hsp70-chaperone-accelerated substrate folding. To see this figure in color, go online.

binding and ATP hydrolysis, such an unfoldase mechanism can be indirectly demonstrated by comparing the conformational distributions of the substrate proteins bound in the closed state of Hsp70 and those of the isolated substrate proteins. The results showed that compared to the isolated substrate, which samples both folded conformations and misfolded conformations (Fig. 2, *A* and *B*; Fig. S8), the substrate bound in the closed state of Hsp70 dominantly adopts the fully unfolded conformations lacking misfolded structural features (Fig. 4, *E* and *F*; Fig. S8). Such differences may suggest that Hsp70 has the ability to unfold the misfolded protein, supporting the unfoldase mechanism. However, this kind of simulation cannot unambiguously discriminate whether the Hsp70-induced substrate unfolding occurs by a power-stroke mechanism or other mechanisms.

In this work, the release of the substrate from the Hsp70 chaperone was not explicitly modeled. Instead, the Hsp70 chaperone was removed once the substrate folds to a near-native structure. Although it is still in debate whether the substrate release follows an active mechanism or passive mechanism (66), the interaction strength between the substrate protein and the Hsp70 chaperone can affect the lifetime of the bound state. Based on the above discussions, the interaction strength cannot be too weak. Otherwise, the substrate cannot remain bound with the chaperone during folding. On the other hand, overstabilization of the bound state may slow down the substrate release (64), which in turn hinders the functional cycle of the chaperones. In future studies, it will be interesting to explicitly simulate the substrate release step and investigate how the binding affinity between the chaperone and the substrate protein can affect the efficiency of the chaperone actions.

SUPPORTING MATERIAL

Supporting material can be found online at https://doi.org/10.1016/j.bpj.2021.03.013.

AUTHOR CONTRIBUTIONS

J.L., W.L., and W.W. designed the research. J.L. and X.Z. carried out simulations. J.L., X.Z., Y.W., Y.S., W.L., and W.W. analyzed the data. J.L., W.L., and W.W. wrote the article.

ACKNOWLEDGMENTS

The authors acknowledge support from the National Natural Science Foundation of China (Nos. 11974173, 11934008, and 11874197) and the HPC center of Nanjing University.

SUPPORTING CITATIONS

Reference (67) appears in the Supporting material.

REFERENCES

1. Ellis, R. J. 2001. Macromolecular crowding: obvious but underappreciated. *Trends Biochem. Sci.* 26:597–604.
2. Fink, A. L. 1999. Chaperone-mediated protein folding. *Physiol. Rev.* 79:425–449.

3. Young, J. C., V. R. Agashe, …, F. U. Hartl. 2004. Pathways of chaperone-mediated protein folding in the cytosol. *Nat. Rev. Mol. Cell Biol.* 5:781–791.

4. Chen, Y.-J., and M. Inouye. 2008. The intramolecular chaperone-mediated protein folding. *Curr. Opin. Struct. Biol.* 18:765–770.

5. Kim, Y. E., M. S. Hipp, …, F. U. Hartl. 2013. Molecular chaperone functions in protein folding and proteostasis. *Annu. Rev. Biochem.* 82:323–355.

6. Zhao, L., G. Vecchi, …, F. U. Hartl. 2019. The Hsp70 chaperone system stabilizes a thermo-sensitive subproteome in E. coli. *Cell Rep.* 28:1335–1345.e6.

7. De Los Rios, P., and A. Barducci. 2014. Hsp70 chaperones are nonequilibrium machines that achieve ultra-affinity by energy consumption. *eLife.* 3:e02218.

8. Clerico, E. M., J. M. Tilitsky, …, L. M. Gierasch. 2015. How Hsp70 molecular machines interact with their substrates to mediate diverse physiological functions. *J. Mol. Biol.* 427:1575–1588.

9. Wu, S., L. Hong, …, S. Perrett. 2020. Kinetics of the conformational cycle of Hsp70 reveals the importance of the dynamic and heterogeneous nature of Hsp70 for its function. *Proc. Natl. Acad. Sci. USA.* 117:7814–7823.

10. Stan, G., B. R. Brooks, and D. Thirumalai. 2005. Probing the "annealing" mechanism of GroEL minichaperone using molecular dynamics simulations. *J. Mol. Biol.* 350:817–829.

11. Kmiecik, S., and A. Kolinski. 2011. Simulation of chaperonin effect on protein folding: a shift from nucleation-condensation to framework mechanism. *J. Am. Chem. Soc.* 133:10283–10289.

12. Chakrabarti, S., C. Hyeon, …, D. Thirumalai. 2017. Molecular chaperones maximize the native state yield on biological times by driving substrates out of equilibrium. *Proc. Natl. Acad. Sci. USA.* 114:E10919–E10927.

13. Goloubinoff, P., A. S. Sassi, …, P. De Los Rios. 2018. Chaperones convert the energy from ATP into the nonequilibrium stabilization of native proteins. *Nat. Chem. Biol.* 14:388–395.

14. Ellis, R. J. 1994. Molecular chaperones. Opening and closing the Anfinsen cage. *Curr. Biol.* 4:633–635.

15. Todd, M. J., G. H. Lorimer, and D. Thirumalai. 1996. Chaperonin-facilitated protein folding: optimization of rate and yield by an iterative annealing mechanism. *Proc. Natl. Acad. Sci. USA.* 93:4030–4035.

16. Lin, Z., and H. S. Rye. 2006. GroEL-mediated protein folding: making the impossible, possible. *Crit. Rev. Biochem. Mol. Biol.* 41:211–239.

17. Weissman, J. S., Y. Kashi, …, A. L. Horwich. 1994. GroEL-mediated protein folding proceeds by multiple rounds of binding and release of nonnative forms. *Cell.* 78:693–702.

18. Gulukota, K., and P. G. Wolynes. 1994. Statistical mechanics of kinetic proofreading in protein folding in vivo. *Proc. Natl. Acad. Sci. USA.* 91:9292–9296.

19. Brinker, A., G. Pfeifer, …, M. Hayer-Hartl. 2001. Dual function of protein confinement in chaperonin-assisted protein folding. *Cell.* 107:223–233.

20. Kityk, R., J. Kopp, …, M. P. Mayer. 2012. Structure and dynamics of the ATP-bound open conformation of Hsp70 chaperones. *Mol. Cell.* 48:863–874.

21. Zahn, M., N. Berthold, …, N. Sträter. 2013. Structural studies on the forward and reverse binding modes of peptides to the chaperone DnaK. *J. Mol. Biol.* 425:2463–2479.

22. Nishikawa, T., A. Nagadoi, …, Y. Nishimura. 1998. Solution structure of the DNA-binding domain of human telomeric protein, hTRF1. *Structure.* 6:1057–1065.

23. Goloubinoff, P., and P. De Los Rios. 2007. The mechanism of Hsp70 chaperones: (entropic) pulling the models together. *Trends Biochem. Sci.* 32:372–380.

24. Sharma, S. K., P. De los Rios, …, P. Goloubinoff. 2010. The kinetic parameters and energy cost of the Hsp70 chaperone as a polypeptide unfoldase. *Nat. Chem. Biol.* 6:914–920.

25. Sekhar, A., R. Rosenzweig, …, L. E. Kay. 2016. Hsp70 biases the folding pathways of client proteins. *Proc. Natl. Acad. Sci. USA.* 113:E2794–E2801.

26. Jewett, A. I., and J. E. Shea. 2010. Reconciling theories of chaperonin accelerated folding with experimental evidence. *Cell. Mol. Life Sci.* 67:255–276.

27. England, J., D. Lucent, and V. Pande. 2008. Rattling the cage: computational models of chaperonin-mediated protein folding. *Curr. Opin. Struct. Biol.* 18:163–169.

28. Jewett, A. I., A. Baumketner, and J.-E. Shea. 2004. Accelerated folding in the weak hydrophobic environment of a chaperonin cavity: creation of an alternate fast folding pathway. *Proc. Natl. Acad. Sci. USA.* 101:13192–13197.

29. Takagi, F., N. Koga, and S. Takada. 2003. How protein thermodynamics and folding mechanisms are altered by the chaperonin cage: molecular simulations. *Proc. Natl. Acad. Sci. USA.* 100:11367–11372.

30. Betancourt, M. R., and D. Thirumalai. 1999. Exploring the kinetic requirements for enhancement of protein folding rates in the GroEL cavity. *J. Mol. Biol.* 287:627–644.

31. Chan, H. S., and K. A. Dill. 1996. A simple model of chaperonin-mediated protein folding. *Proteins.* 24:345–351.

32. Xu, W.-X., J. Wang, and W. Wang. 2005. Folding behavior of chaperonin-mediated substrate protein. *Proteins.* 61:777–794.

33. Sekhar, A., R. Rosenzweig, …, L. E. Kay. 2015. Mapping the conformation of a client protein through the Hsp70 functional cycle. *Proc. Natl. Acad. Sci. USA.* 112:10395–10400.

34. Lee, J. H., D. Zhang, …, S. Cavagnero. 2015. Heterogeneous binding of the SH3 client protein to the DnaK molecular chaperone. *Proc. Natl. Acad. Sci. USA.* 112:E4206–E4215.

35. Sekhar, A., M. Santiago, …, S. Cavagnero. 2012. Transient interactions of a slow-folding protein with the Hsp70 chaperone machinery. *Protein Sci.* 21:1042–1055.

36. Terakawa, T., and S. Takada. 2011. Multiscale ensemble modeling of intrinsically disordered proteins: p53 N-terminal domain. *Biophys. J.* 101:1450–1458.

37. Kenzaki, H., N. Koga, …, S. Takada. 2011. CafeMol: a coarse-grained biomolecular simulator for simulating proteins at work. *J. Chem. Theory Comput.* 7:1979–1989.

38. Clementi, C., H. Nymeyer, and J. N. Onuchic. 2000. Topological and energetic factors: what determines the structural details of the transition state ensemble and "en-route" intermediates for protein folding? An investigation for small globular proteins. *J. Mol. Biol.* 298:937–953.

39. Go, N. 1983. Theoretical studies of protein folding. *Annu. Rev. Biophys. Bioeng.* 12:183–210.

40. Onuchic, J. N., Z. Luthey-Schulten, and P. G. Wolynes. 1997. Theory of protein folding: the energy landscape perspective. *Annu. Rev. Phys. Chem.* 48:545–600.

41. Shea, J. E., J. N. Onuchic, and C. L. Brooks, III. 1999. Exploring the origins of topological frustration: design of a minimally frustrated model of fragment B of protein A. *Proc. Natl. Acad. Sci. USA.* 96:12512–12517.

42. Takada, S. 2019. Gō model revisited. *Biophys. Physicobiol.* 16:248–255.

43. Bertelsen, E. B., L. Chang, …, E. R. P. Zuiderweg. 2009. Solution conformation of wild-type *E. coli* Hsp70 (DnaK) chaperone complexed with ADP and substrate. *Proc. Natl. Acad. Sci. USA.* 106:8471–8476.

44. Li, W., H. Yoshii, …, S. Takada. 2010. Multiscale methods for protein folding simulations. *Methods.* 52:106–114.

45. Li, W., P. G. Wolynes, and S. Takada. 2011. Frustration, specific sequence dependence, and nonlinearity in large-amplitude fluctuations of allosteric proteins. *Proc. Natl. Acad. Sci. USA.* 108:3504–3509.

46. Li, W., W. Wang, and S. Takada. 2014. Energy landscape views for interplays among folding, binding, and allostery of calmodulin domains. *Proc. Natl. Acad. Sci. USA.* 111:10550–10555.

47. Takada, S. 2012. Coarse-grained molecular simulations of large biomolecules. *Curr. Opin. Struct. Biol.* 22:130–137.

48. Kim, Y. C., and G. Hummer. 2008. Coarse-grained models for simulations of multiprotein complexes: application to ubiquitin binding. *J. Mol. Biol.* 375:1416–1433.

49. Jackson, M. B. 2006. Molecular and Cellular Biophysics. Cambridge University Press, Cambridge, UK.

50. Zhuravleva, A., E. M. Clerico, and L. M. Gierasch. 2012. An interdomain energetic tug-of-war creates the allosterically active state in Hsp70 molecular chaperones. *Cell.* 151:1296–1307.

51. Mayer, M. P., and B. Bukau. 2005. Hsp70 chaperones: cellular functions and molecular mechanism. *Cell. Mol. Life Sci.* 62:670–684.

52. Okazaki, K., N. Koga, …, P. G. Wolynes. 2006. Multiple-basin energy landscapes for large-amplitude conformational motions of proteins: structure-based molecular dynamics simulations. *Proc. Natl. Acad. Sci. USA.* 103:11844–11849.

53. Tanaka, T., N. Hori, and S. Takada. 2015. How co-translational folding of multi-domain protein is affected by elongation schedule: molecular simulations. *PLoS Comput. Biol.* 11:e1004356.

54. Humphrey, W., A. Dalke, and K. Schulten. 1996. VMD: visual molecular dynamics. *J. Mol. Graph.* 14:33–38, 27–28..

55. DeLano, W. L. 2009. The PyMOL Molecular Graphics System. DeLano Scientific, San Carlos, CA.

56. Böcking, T., F. Aguet, …, T. Kirchhausen. 2011. Single-molecule analysis of a molecular disassemblase reveals the mechanism of Hsc70-driven clathrin uncoating. *Nat. Struct. Mol. Biol.* 18:295–301.

57. Priya, S., S. K. Sharma, and P. Goloubinoff. 2013. Molecular chaperones as enzymes that catalytically unfold misfolded polypeptides. *FEBS Lett.* 587:1981–1987.

58. Morán Luengo, T., R. Kityk, …, S. G. D. Rüdiger. 2018. Hsp90 breaks the deadlock of the Hsp70 chaperone system. *Mol. Cell.* 70:545–552.e9.

59. Schlecht, R., A. H. Erbse, …, M. P. Mayer. 2011. Mechanics of Hsp70 chaperones enables differential interaction with client proteins. *Nat. Struct. Mol. Biol.* 18:345–351.

60. Mashaghi, A., S. Bezrukavnikov, …, S. J. Tans. 2016. Alternative modes of client binding enable functional plasticity of Hsp70. *Nature.* 539:448–451.

61. Imamoglu, R., D. Balchin, …, F. U. Hartl. 2020. Bacterial Hsp70 resolves misfolded states and accelerates productive folding of a multidomain protein. *Nat. Commun.* 11:365.

62. Gray, T. E., J. Eder, …, A. R. Fersht. 1993. Refolding of barnase mutants and pro-barnase in the presence and absence of GroEL. *EMBO J.* 12:4145–4150.

63. Libich, D. S., V. Tugarinov, and G. M. Clore. 2015. Intrinsic unfoldase/foldase activity of the chaperonin GroEL directly demonstrated using multinuclear relaxation-based NMR. *Proc. Natl. Acad. Sci. USA.* 112:8817–8823.

64. Wu, K., F. Stull, …, J. C. A. Bardwell. 2019. Protein folding while chaperone bound is dependent on weak interactions. *Nat. Commun.* 10:4833.

65. Horowitz, S., P. Koldewey, …, J. C. A. Bardwell. 2018. Folding while bound to chaperones. *Curr. Opin. Struct. Biol.* 48:1–5.

66. Yang, J., Y. Zong, …, Q. Liu. 2017. Conformation transitions of the polypeptide-binding pocket support an active substrate release from Hsp70s. *Nat. Commun.* 8:1201.

67. Spudich, G. M., E. J. Miller, and S. Marqusee. 2004. Destabilization of the *Escherichia coli* RNase H kinetic intermediate: switching between a two-state and three-state folding mechanism. *J. Mol. Biol.* 335:609–618.

Biophysical Journal, Volume *120*

Supplemental information

Energy landscape remodeling mechanism of Hsp70-chaperone-accelerated protein folding

Jiajun Lu, Xiaoyi Zhang, Yichao Wu, Yuebiao Sheng, Wenfei Li, and Wei Wang

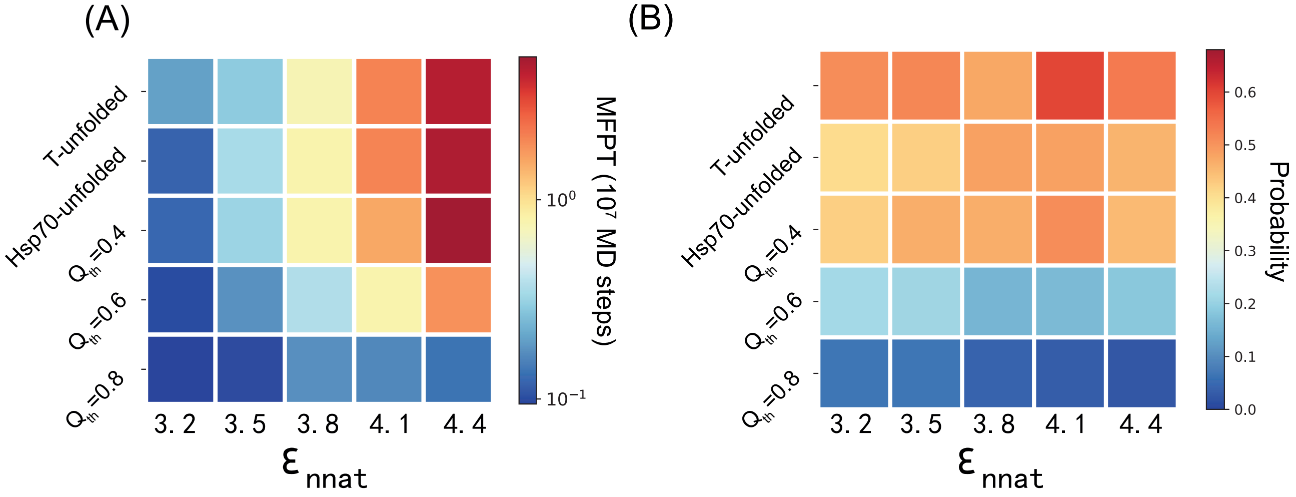

Figure S1. (MFPT of substrate hTRF1 folding (A) and populations of the frustrated folding pathway (B) calculated based on different simulation strategies and frustration extents for the hTRF1 with two pairs of non-native contacting interactions. T-unfolded: spontaneous folding starting from the initial structures prepared by thermal denaturation; Hsp70-unfolded: spontaneous folding starting from the initial structures prepared by unfolding action of the Hsp70; Q_{th} = 0.4, 0.6, and 0.8: Hsp70 mediated folding with the chaperone being removed when the Q score exceeds 0.4, 0.6, and 0.8, respectively.

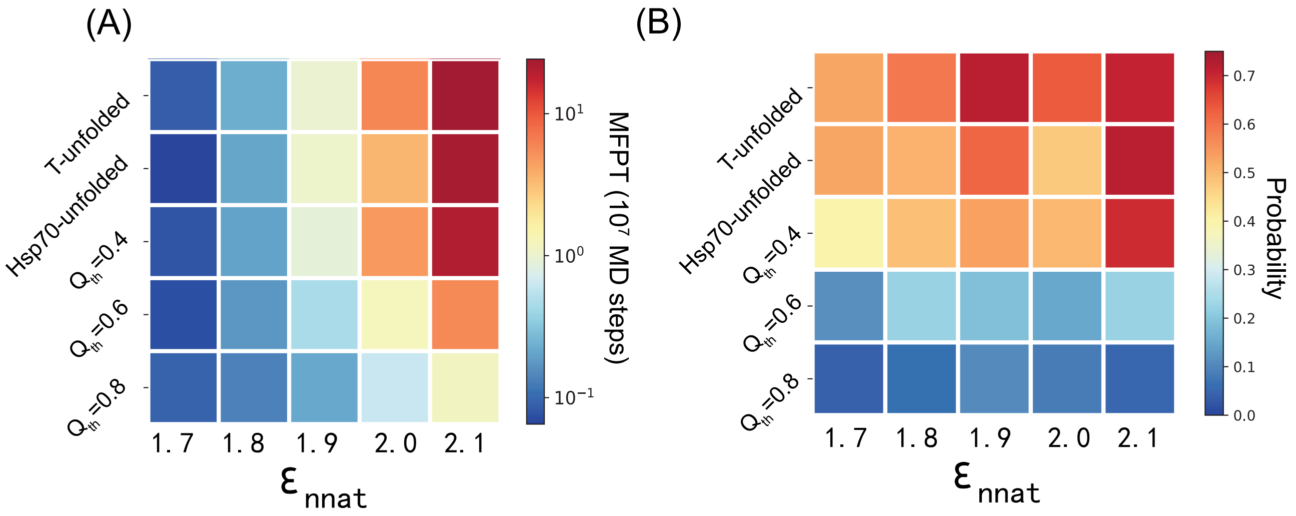

Figure S2. MFPT of substrate hTRF1 folding (A) and populations of the frustrated folding pathway (B) calculated based on different simulation strategies and frustration extents for the hTRF1 with 25 pairs of non-native contacting interactions.

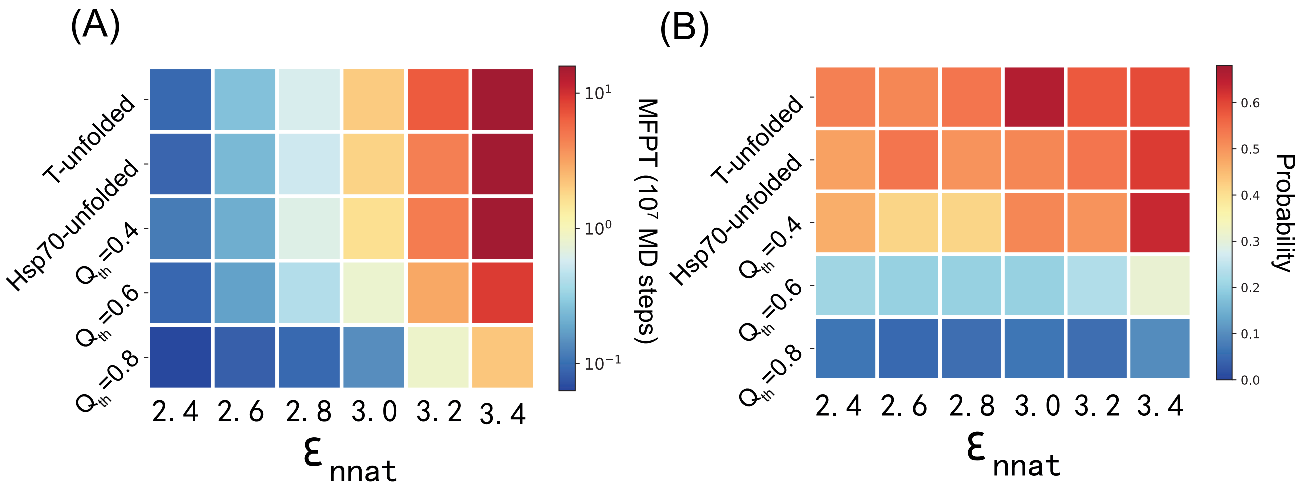

Figure S3. MFPT of substrate hTRF1 folding (A) and populations of the frustrated folding pathway (B) calculated based on different simulation strategies and frustration extents for the hTRF1 with the HP parameter C_{HP} set as 4.0.

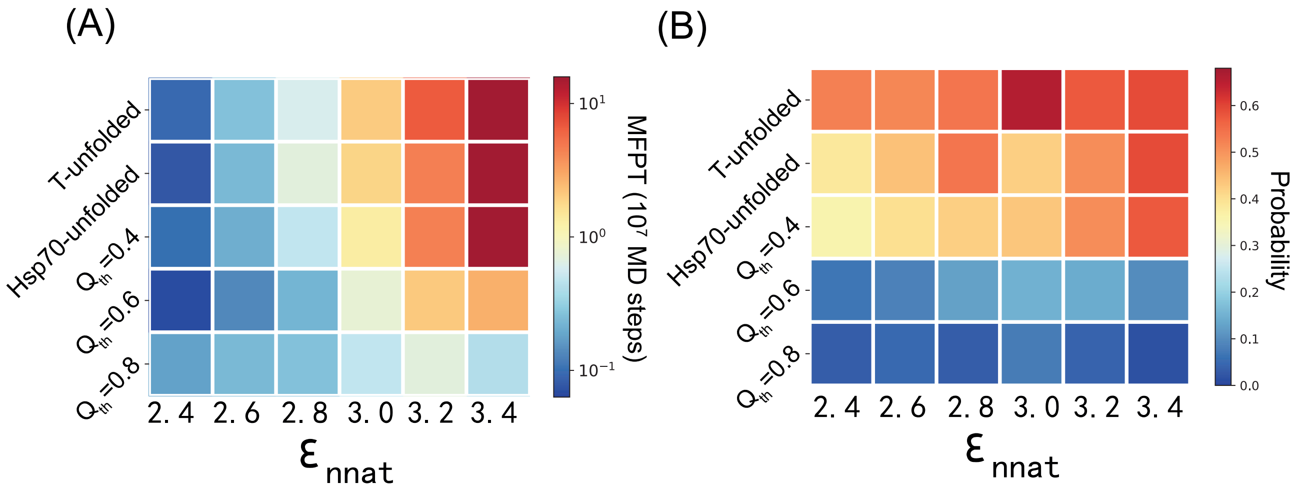

Figure S4. MFPT of substrate hTRF1 folding (A) and populations of the frustrated folding pathway (B) calculated based on different simulation strategies and frustration extents for the hTRF1 with the C_{HP} set as 6.0.

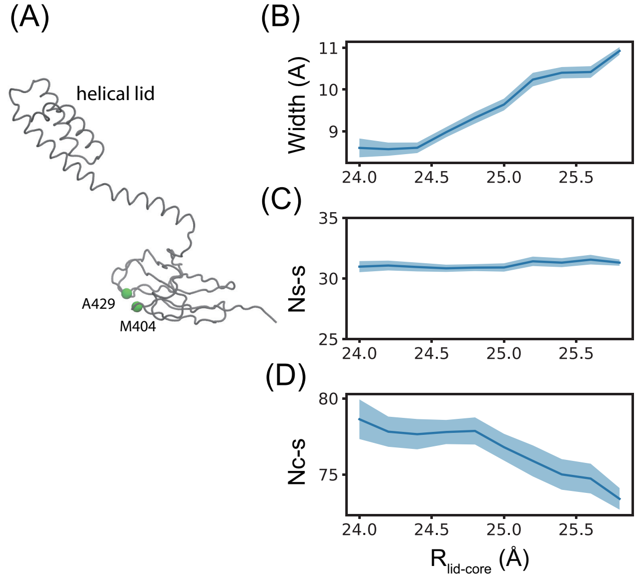

Figure S5. Effect of closed to open conformational change on the folding of the substrate hTRF1. (A) Illustrating structure of the substrate binding domain(SBD) of the Hsp70 with open conformation. (B) Average width of the substrate binding groove as a function of the reaction coordinate $R_{\mathrm{lid-core}}$. Here the reaction coordinate $R_{\mathrm{lid-core}}$ is defined by the distance between the centers of mass of the helical lid (S504 to A602) and the substrate binding core (V389 to A503) of the SBD, which was used to monitor the closed to open conformational change of the Hsp70. The width of the substrate binding groove was calculated based on the two labelled residues. (B) Average total number of the formed intra-molecule contacts for the substrate hTRF1. (C) The average number of the formed inter-molecule contacts between the substrate and the Hsp70.

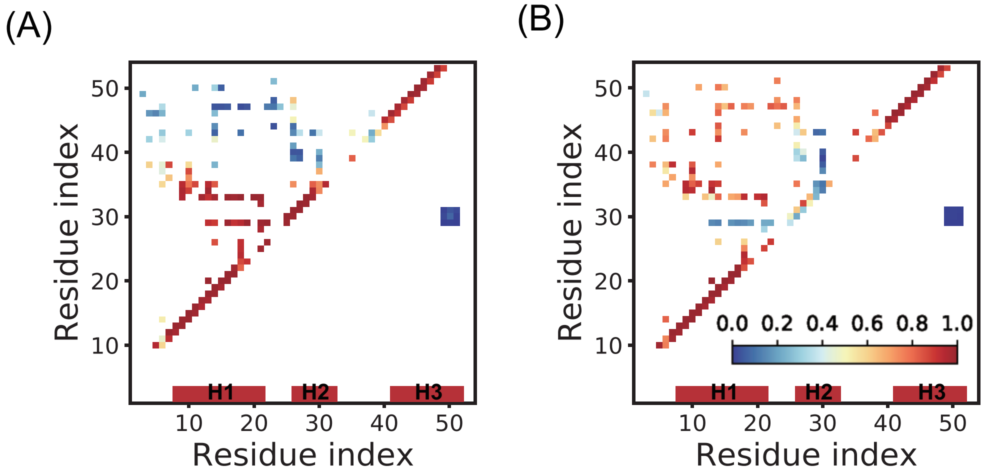

Figure S6. Structure features of the transition state ensembles of hTRF1 folding. (A,B) Probabilities of the contact formation at the transition state ensembles based on the simulations without (A) and with (B) the presence of the Hsp70 chaperone. The results for the native contacts are shown in the upper-left part, and the results for the nine residue pairs with non-native contacting interactions are shown in the lower-right part. One can see that the Hsp70 interactions tend to modulate the transition state structure of the substrate folding by favoring the formation of the native contacts between the H1 and H3.

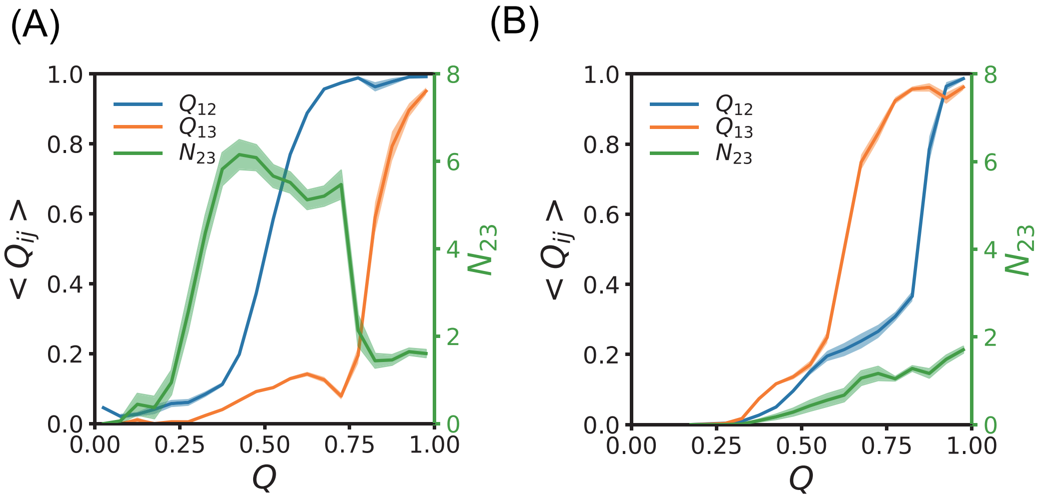

Figure S7. The average fraction of formed native contacts between the three helices (Q_{12}, Q_{13}) and the total number of the formed contacts between the H2 and H3 (N_{23}, right axis) as a function of the fraction of the formed total native contacts Q for the simulations of hTRF1 folding without (A) and with (B) the Hsp70 chaperon. Without Hsp70, the contacts between the H1 and H2 forms much earlier than those between the H1 and H3. Whereas with the presence of the Hsp70, the H1-H3 contacts (Q_{13}) can form at much earlier stage of the folding. Such results suggest that the Hsp70 tends to increase the population of the pathway via I_{13}. Consistent with the discussions in the main text, the formation of the non-native contacts between the H2 and H3 is much reduced with the presence of the Hsp70.

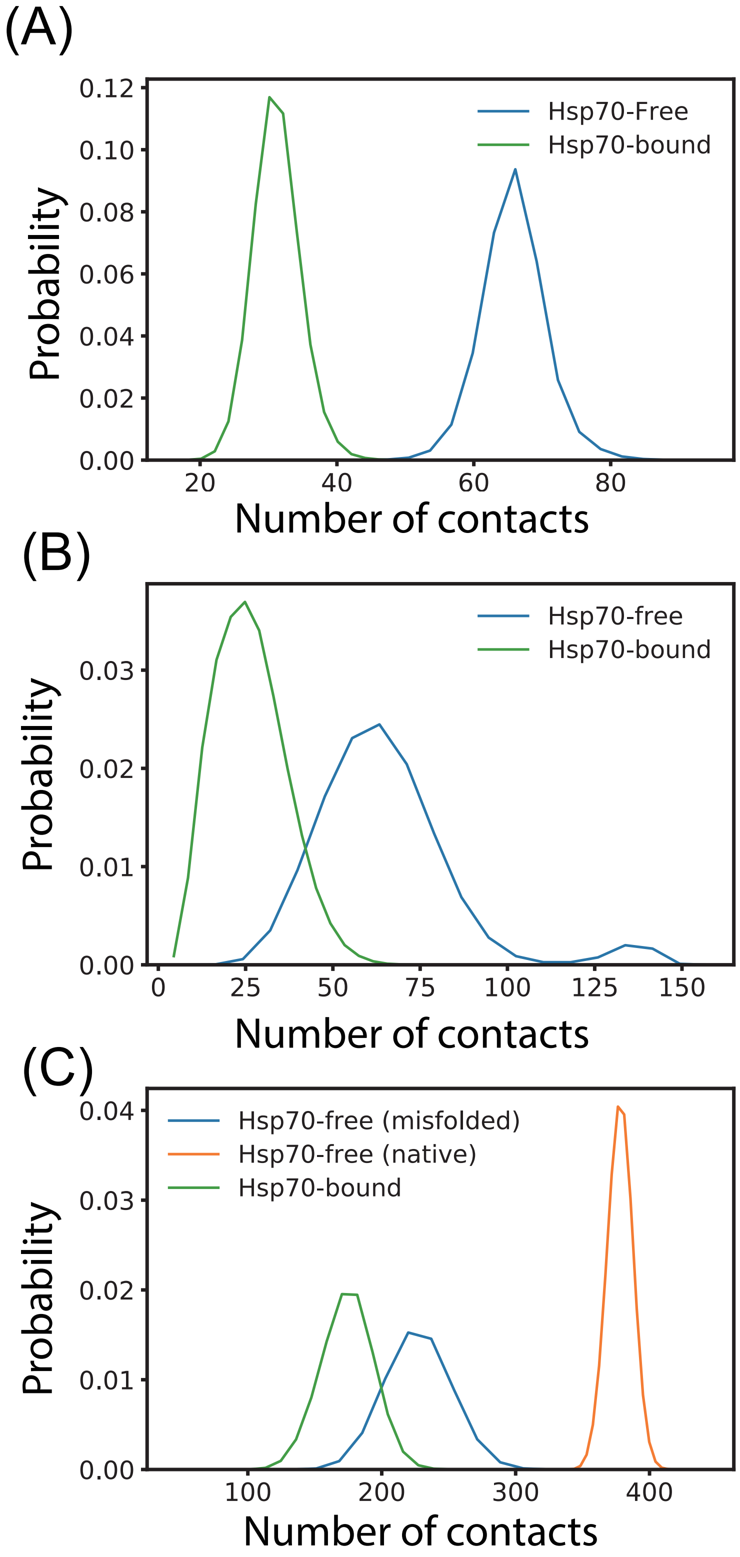

Figure S8. The distributions of the total intra-molecule contact numbers for substrate proteins hTRF1(A), SH3 (B), and RNase H (C) without Hsp70 binding (blue) and with the substrate bound to the closed form of the Hsp70 (green). The total contacts for the native state (blue) and misfolded state (orange) of the substrate RNase H were shown separately in (C).

Table S1. The scaling factor λ_{SB} controlling the interaction strengths of the structure-based interactions for the substrate proteins hTRF1, SH3, and RNase H. The ΔG_{NU} (or the population of unfolded state P_{U}) from simulations and experimental measurements (1–3).

Substrate	λ_{SB}	$P_{\mathrm{U}}^{\mathrm{simu.}}$	$P_{\mathrm{U}}^{\mathrm{exp.}}$
hTRF1	0.66	1%	1%
SH3	0.648	35%	38%
Substrate	λ_{SB}	$\Delta G_{UN}^{\mathrm{simu.}}$	$\Delta G_{\mathrm{UN}}^{\mathrm{exp.}}$
RNase H	0.60	10.2	9.7

Table S2. The residue pairs involved in the non-native contacting interactions for the three substrate proteins. The non-native contacting interactions were introduced for nine residue pairs.

hTRF1	I29-T49	I29-M50	I29-K51	I30-T49	I30-M50	I30-K51	L31-T49	L31-M50	L31-K51
SH3	A11-Q23	A11-I24	A11-L25	T12-Q23	T12-I24	T12-L25	A13-Q23	A13-I24	A13-L25
RNase H	I25-A109	I25-A110	I25-L111	L26-A109	L26-A110	L26-L111	R27-A109	R27-A110	R27-L111

Table S3. The residue pairs involved in the non-native contacting interactions for the hTRF1. The non-native contacting interactions were introduced for 2 or 25 residue pairs.

2 pairs	I30-M50	L31-M50							
25 pairs	K28-R48	K28-T49	K28-M50	K28-K51	K28-K52	I29-R48	I29-T49	I29-M50	I29-K51
	I29-K52	L30-R48	L30-T49	L30-M50	L30-K51	L30-K52	L31-R48	L31-T49	L31-M50
	L31-K51	L31-K52	H32-R48	H32-T49	H32-M50	H32-K51	H32-K52		

Table S4. The parameters set ΔV_s and Δ_s corresponding to the ADP state (closed conformation is much more stable than the open conformation) and ATP state (open conformation is much more stable than the closed conformation).

State	ΔV_s	Δ_s
ADP	-60	125
ATP	35	125

Table S5. The parameters used in the hydrophobic interactions for the chaperone-substrate interactions(4)

Amino acid	Ala	Arg	Asn	Asp	Cys	Gln	Glu	Gly	His	Ile
$\epsilon_{\mathrm{HP}}^{A}$	0.42	-1.37	-0.82	-1.05	2.09	-0.30	-0.87	0.00	0.18	2.45
Amino acid	Leu	Lys	Met	Phe	Pro	Ser	Thr	Trp	Tyr	Val
$\epsilon_{\mathrm{HP}}^{A}$	2.31	-1.35	1.67	2.43	0.98	-0.05	0.35	3.06	1.31	1.66

Table S6. The parameters used in the excluded volume interactions for the chaperone-substrate interactions taken from Ref. (5). The original values were scaled by 0.9.

Amino acid	Ala	Arg	Asn	Asp	Cys	Gln	Glu	Gly	His	Ile
σ(Å)	4.50	5.94	5.13	5.04	4.95	5.40	5.31	4.05	5.49	5.58
Amino acid	Leu	Lys	Met	Phe	Pro	Ser	Thr	Trp	Tyr	Val
σ(Å)	5.58	5.76	5.58	5.76	5.04	4.68	5.04	6.12	5.85	5.31

Supporting References

1. A Sekhar, R Rosenzweig, G Bouvignies, LE Kay, Mapping the conformation of a client protein through the hsp70 functional cycle. *Proc. Natl. Acad. Sci. U.S.A.* **112**, 10395–10400 (2015).
2. JH Lee, et al., Heterogeneous binding of the sh3 client protein to the dnak molecular chaperone. *Proc. Natl. Acad. Sci. U.S.A.* **112**, E4206–E4215 (2015).
3. GM Spudich, EJ Miller, S Marqusee, Destabilization of the escherichia coli rnase h kinetic intermediate: switching between a two-state and three-state folding mechanism. *J. Mol. Biol.* **335**, 609–618 (2004).
4. T Tanaka, N Hori, S Takada, How co-translational folding of multi-domain protein is affected by elongation schedule: Molecular simulations. *PLOS Comput. Biol.* **11** (2015).
5. YC Kim, G Hummer, Coarse-grained models for simulations of multiprotein complexes: application to ubiquitin binding. *J. Mol. Biol.* **375**, 1416–1433 (2008).
